# Supplementary material for: Bristol girls dance project feasibility study: using a pilot economic evaluation to inform design of a full trial
Source: BMJ Open. 2013 Dec 19;3(12):e003726. doi: 10.1136/bmjopen-2013-003726 (PMC3884756; doi:10.1136/bmjopen-2013-003726)
Supplement: Author's manuscript [file bmjopen-2013-003726.draft_revisions.pdf]

Bristol Girls Dance Project feasibility study: using a pilot economic evaluation to inform design of a full trial

Journal:	*BMJ Open*
Manuscript ID:	bmjopen-2013-003726
Article Type:	Research
Date Submitted by the Author:	07-Aug-2013

Complete List of Authors:	Powell, Jane; University of the West of England, Bristol, Health and Social Sciences<br>Carroll, Fran; University of Bristol, School of Social and Community Medicine<br>Sebire, Simon; University of Bristol, Centre for Exercise Nutrition and Health Sciences<br>Haase, Anne; University of Bristol, Centre for Exercise Nutrition and Health Sciences<br>Jago, Russ; University of Bristol, Centre for Exercise, Nutrition and Health Sciences

**Primary Subject Heading**:	Public health
Secondary Subject Heading:	Health economics, Sports and exercise medicine
Keywords:	SPORTS MEDICINE, HEALTH ECONOMICS, PUBLIC HEALTH

SCHOLARONE™
Manuscripts

RESEARCH PILOT STUDY

Bristol Girls Dance Project feasibility study: using a pilot economic evaluation to inform design of a full trial

Powell, J.[1§] Carroll, FE.[2] Sebire SJ.[3] Haase, AM.[3] Jago, R.[3]

[1] Professor of Public Health Economics, Department of Health and Applied Social Sciences, University of the West of England, Glenside Campus, Stapleton, Bristol, BS16 1DD, UK.

[2]Dr Fran E Carroll, Research Assistant, School of Social and Community Medicine, University of Bristol, BS8 2PS, UK.

[3]Dr Simon J Sebire, Post-Doctoral Researcher, Centre for Exercise Nutrition and Health Sciences, School for Policy Studies, University of Bristol, BS8 1TZ, UK.

[3]Dr Anne M Haase, Senior Lecturer in Exercise, Nutrition & Health, Centre for Exercise, Nutrition and Health Sciences, School for Policy Studies, University of Bristol, BS8 1TZ, UK.

[3]Professor Russell Jago, Professor of Paediatric Physical Activity & Public Health Exercise, Nutrition & Health Sciences, Centre for Exercise, Nutrition & Health Sciences, School for Policy Studies, University of Bristol, BS8 1TZ, UK.

[§]Corresponding author Professor Jane Powell - jane.powell@uwe.ac.uk, Department of Health and Applied Social Sciences, University of the West of England, Bristol, BS16 1DD, UK. 0044 117 3288752

For peer review only

ABSTRACT

Wider context: There is currently little guidance for pilot trial economic evaluation where health outcomes and costs are influenced by a range of wider determinants and factors.

Objectives: This paper presents the findings of a pilot economic evaluation study running alongside the Bristol Girls Dance Project (BGDP) feasibility study and describes how these have impacted the conduct and design of main trial economic evaluation.

Design: Three-arm, cluster randomised, controlled pilot trial and economic evaluation. Seven schools (n=210) from the Bristol and greater Bristol area, UK were randomly allocated to the intervention arm 3 schools (n= 90) and the control arms 4 schools (n=120).

Intervention: Girls aged 11-12 years with parental consent were provided with two, 90-minute dance sessions per week for 9-weeks at school facilities.

Economic measures: Programme costs, girls' preferences for attributes of dance and competing leisure time activities were measured. Primary outcome: accelerometer-derived moderate-to-vigorous physical activity (MVPA) minutes per day.

Results: After-school dance classes for girls aged 11-12 years are valued favourably in terms of other competing leisure choices compared with older adolescents. Discrete Choice Experiment methods are acceptable to girls of this age. The average cost of the BDGP programme per school not including developmental and research costs at mainstream implementation is $2,555; £1,596; €1,870 and per participant $85.17; £53.21; €62.32 in 2010-11 prices.

Conclusions: The feasibility of providing full cost data for a full trial of the BGDP programme has been established and an embryonic resource use checklist developed for this purpose. This pilot study has demonstrated resources used to develop, prepare and deliver the

For peer review only

programme should be categorised separately, so the cost of the mainstream programme can be estimated accurately in a full trial. A social model of costing that reflects opportunity costs of participants and parents could be considered.

BACKGROUND

Recent influential studies attach substantial economic and social costs to obesity prevalence projections.[1 2] These forecasts are based upon a body of research from long term cohort studies which suggest that change in the prevalence of obesity in children and adolescents born at the millennium is likely to lead to increased health risks in middle-age irrespective of adult adiposity.[3 4 5] Consequently, there is a need for new interventions that focus on preventing obesity or changing diet or physical activity; the two behaviours that are central to the accrual of body mass.

As well as being a health and wellbeing issue, children's obesity also has serious economic impacts. Scarce resources with competing uses in all health systems and the need to decide between new, 'efficacious' primary prevention physical activity programme interventions on the grounds of cost-effectiveness, has increased the significance of economic evaluation as a concept and methodology. Recent guidance from the UK Medical Research Council (MRC) for the development and evaluation of complex behavioural interventions suggests that efficacy and cost-effectiveness should be established before programmes are implemented at the population level.[6 7] However, the meaningful determination of these criteria is often problematic in primary prevention and guidelines for the design and conduct of economic evaluation of complex interventions are at an early stage of development.[8 9 10 11] It is therefore important to develop the conceptual and measurement process by which effectiveness and

For peer review only

cost-effectiveness of complex physical activity interventions can be evaluated in a full trial using a pilot study.

The Bristol Girls Dance Project (BGDP) feasibility trial, examined the potential utility of an after-school dance programme as a means of increasing moderate to vigorous physical activity (MVPA) among 11-12 year old girls (school year 7) seeking to discover any improvements that might need to be made to the programme prior to a main trial.[12] This suggested that it is feasible to deliver the intervention and that participating in dance has the potential to yield change in girls' physical activity, but a larger randomised controlled trial (RCT) would be needed to fully evaluate its effectiveness and cost-effectiveness. In the absence of other evidence for the outcome of dance interventions, another aim of the feasibility pilot was to refine the information required to sufficiently power a full trial.

Preferences for competing after-school activities are potential determinants of the economic benefit of dance intervention, as increased physical activity must be valued in order for it to be maintained [8] and to have potential for long-term impact on physical activity levels. In this study, DCE and survey methods are applied to examine two separate, but complementary aspects of value –preferences for the attributes of dance classes and preferences for dance among other competing alternatives for spending leisure time respectively. Physical activity levels decline during youth [13] with the start of secondary school being a critical period of change, so it was important to establish comparative preferences for after-school, leisure activities on weekdays.

Value is a concept germane to recruitment and retention rates and linked to the outcome dimension of the BGDP intervention and therefore important to examine in detail. Discrete Choice Experiment (DCE) works on the premise that any 'product', for example a healthcare treatment or physical activity programme, can be described by its characteristics, or

For peer review only

attributes, and the extent to which an individual values a 'product' is dependent on the level of these characteristics. [14 15 16]

Thus, this paper reports the learning from and findings of a pilot economic evaluation of the Bristol Girls Dance Project for girls aged 11-12 years in a primary school setting in England impact the design and conduct of a full trial economic evaluation. Other findings from the Bristol Girls Dance Project Feasibility Trial concerning process evaluation, outcomes and effectiveness have been reported elsewhere.[12]

ARTICLE SUMMARY

Article focus

To examine the feasibility of providing complete programme cost data from a funder perspective.

To demonstrate the appropriateness of using DCE and survey methods to derive participants' preferences for dance among other competing leisure activities.

To present learning from reflections on the findings of pilot economic evaluation to inform a full trial economic evaluation

Key messages

A pilot economic evaluation has been applied to learn more about the feasibility of costing the BGDP complex intervention and categorising programme resources to identify the mainstream cost of the intervention during a full trial.

Pilot data from seven schools has been analysed to understand girls' preferences for dance versus other ways of spending their leisure time at age 11-12 years that can help to

For peer review only

conceptualise and measure the outcome dimensions of economic evaluation alongside a full trial.

Strengths and limitations of this study

This pilot study used a systematic approach to consider how the conceptual and measurement process of an economic evaluation might be enhanced in a full trial by learning more about the cost and outcome dimensions of economic evaluation and has produced findings that can be applied to design and conduct an economic evaluation alongside a full cluster RCT.

This feasibility and exploratory pilot study is powered to test the intervention concept, the feasibility of obtaining programme cost data in categories and the evidence required to power a full cluster RCT in the future. Consequently, the variation in programme costs at the school level has not been captured and this is a limitation of the programme cost estimates presented.

METHODS

Bristol Girls Dance Project (BGDP) feasibility study

BGDP was a three-arm, parallel group, cluster randomised, controlled pilot trial with schools as the unit of allocation. Seven schools from three school districts, Bristol, Bath and South Gloucestershire (UK) were recruited to take part in the study from schools in these districts with no current after school dance provision.[12] Stratifying by school district, three schools were randomly allocated to the intervention arm (n= 90) and four schools to the two control arms (n=120) and each school was assigned a dance teacher to lead the sessions. Randomisation was conducted by an independent member of the clinical trials unit at Bristol University using computer-generated random sequences and codes for school district and school name.

For peer review only

Sample size

This feasibility study was powered to test the intervention concept and to provide the necessary information to calculate the sample size of a full cluster RCT and economic evaluation of an after school dance programme. The important parameters detection of 10 minutes difference in MVPA per weekday (50 minutes per week) between the intervention and control groups, intra-class correlation for weekday MVPA at time 2 and associated confidence intervals have been reported and profiled in another paper from this study. [12] For practical reasons the sample was limited to thirty girls aged 11-12 years per school. Girls were recruited from each school at random from those with parental consent.

The intervention

Intervention participants were provided with two, 90-minute dance sessions per week for 9-weeks at school facilities. The hip-hop and street dance style of dance to popular music was facilitated by a professional dance teacher.

Primary outcome measure

All participants were asked to wear an Actigraph accelerometer for seven days at baseline (week 0) during the last two weeks of the intervention (weeks 8 or 9) and 3 months after the intervention ended (Week 20).

Economic measures

1) *Resource use-cost*

Resources used at each stage of the BGDP programme were recorded retrospectively by the project team on time-sheets and expense sheets from a public sector perspective. Costing followed a method applied in a study of complex intervention with children in a school

For peer review only

setting (A Stop Smoking in Schools Trial) ASSIST.[17] Stage 0 intervention planning and development costs or costs associated with running the scientific study (e.g., control group incentives for data collection, control school dance workshops or recruitment events) would not recur during mainstream implementation, but these costs are included for completeness.

Expenses including entry incentives, postage and Criminal Records Bureau (CRB) checking were gathered. Travel time was estimated for all staff inputs at the school locations. Girls in the control schools received incremental thank you gifts at each data collection they attended. Space hire did not incur costs, but estimates of the cost of space hire for dance class delivery are included because costs connected with alternative use of space in schools. School overhead and capital costs are not included.

2) *Discrete Choice Experiment (DCE) and survey of preference ranking and use of leisure time*

BGDP formative qualitative work indicated the frequency of after-school dance classes per week; cost per session and how much leisure time is left over on dance class days for other leisure activities are important considerations for girls.[18] Participants were asked to select the ‘dance class scenario’ they preferred from a pair of options. Table I presents the 4 paired scenarios (1A:1B, 2A:2B, 3A:3B, 4A:4B) consisting of a randomly determined combination of three attributes, each with two levels.

Table I

Four paired choice scenarios were administered to 210 girls in seven schools - 3 intervention schools (n=90) and 4 control schools (n=120). Measures were taken at baseline (time 0) and

For peer review only

at 9 weeks (time 1) using large cards, and girls' preferred choice for each pair of scenarios was recorded by the project team. Two time points were needed to establish change in preferences before and after the intervention. Participants were asked to give preference ratings for ten leisure activities on weekdays using a ten-point scale (1 = favourite; 10 = least favourite). Participant responses were collected on Personal Digital Assistants (PDAs) and downloaded to a customised database.

Ethics

Potential participants in all seven schools were told that there was a maximum of 30 randomly assigned spaces at the dance classes. The study was approved by a University of Bristol ethics committee and informed parental consent was obtained for all participants.

Analyses

Proportions of the sample ranking ten weekday leisure activities as first choice activity (rank = 1) were calculated after participants had rated all ten leisure activities from 1 to 10. Responses from participants with repetition of ratings for one or more leisure activities or missing ratings for leisure activities were excluded. Overall, where the proportion of the sample rating activities as their first choice was the same, these activities were assigned the same rank across all ten activities. Valid responses as a proportion of total responses for the survey ranking leisure activities were t2=178/210, t1=130/210, t0=68/210 across all group allocations.

DCE data was 'effects-coded' [19] using STATA [20] and analysed using conditional logistic regression. Effects coding is similar to dummy variable coding, but is preferable in this instance because interaction or trade-off between the attributes is likely to take place as well

For peer review only

as a main effect. The coefficients for each attribute are a measure of the influence of that attribute level on choice. Positive values represent a positive influence on choice, or in other words, a preference for that level of an attribute. These results can be used to establish girls' overall preferences for attributes, as well as the order of their preferences (i.e. which attribute is most and least important). Participants with missing data were excluded from the DCE analysis.

Total and average cost estimates from a funder perspective were identified and derived for BGDP based on staged timing, quantity, frequency and price of resource use in 2010-11 prices.

RESULTS

Identification and timing of resources used

Table II identifies and describes at four stages the resources use of the BGDP programme and presents total cost estimates. The proportion of total costs incurred were 42% at stage 0, 5% at stage 1, 47% at stage 2 and 6% at stage 3. BGDP required at least 286 hours of staff input. Eighteen BGDP dance classes (2 classes per week for 9 weeks) of 90 minutes duration were delivered to 90 girls in three intervention schools (30 per school) for 81 hours (27 hours per school) at a total estimated cost of $7,666; £4,789; €5,609 in 2010-11 prices.[21 22] Removing stage 0 programme development and stage 1-3 research costs revealed an average cost of repeating a BDGP programme in its mainstream form per school of $2,555; £1,596; €1,870 and per participant of $85.17; £53.21; €62.32 in 2010-11 prices. There are no confidence intervals for these average programme cost or participant estimates (see limitations of this pilot study).

For peer review only

Table II here

The BGDP programme compares favourably in terms of average cost per user with the only other evidence for costing an after-school dance class programme in England - estimated by Fordham and Barton [23] and included in NICE guidance [24] on the promotion of physical activity in children. These estimates [23 24] were based on a report of the NRG dance programme [25] which was delivered at a total cost of $32,975; £20,600; €24,129 at 2010-11 prices. Assumptions were made [23] to derive costs for NICE guidance from the NRG report.[25] The shorter and less intensive NRG programme comprised 10 dance classes of 60 minutes duration for an assumed number of 24 girls in 14 groups or schools (n=336 girls) for 140 hours with a cost per participant of $98.14; £61.31; €71.81, in 2010-11 prices. This includes 140 hours of teacher time sourced from national pay scales for England at £23.57 per hour at 2010-11 prices.[26]

Preferences for leisure activities

All girls were asked to rank ten after-school leisure activities by first preference activity. Table III presents proportions across the participants (n=210) for preference ranks for after-school leisure activities for all group allocations at each time point demonstrating consistency in preference ranks indicating girls' selection of first choice leisure activities at each time point. The after-school leisure activities indicating the highest proportion of first choice preference rankings at each time point include 'hanging out with friends away from home just for fun' (Ranking at t2=1, t1=1, t0=2); 'take part in sports, athletics or physical activity' (Ranking at t2=2, t1=2, t0=1) and 'using the internet for fun: chats, YouTube, Facebook, Bebo, Myspace, looking for music' (Ranking at t0=3, t1=3, t2=2).

For peer review only

Table III here

DCE results

Table IV indicates girls in this sample have a preference for "time left for other leisure activities on dance class days", over the "cost of" and "frequency of dance classes per week". Analysis of preference levels within each attribute suggests 2 hours is preferred to 3 hours remaining for other leisure activities on dance class days. Girls were least concerned with the frequency of dance classes per week with preference proportions suggesting 2 classes were preferred to 1 dance class per week. This pattern was consistent in all groups at t0 and t1.

Table IV here

DISCUSSION

What is already known on this topic

There is minimal guidance to support how economic evaluations of complex public health interventions should be designed and conducted in school and community settings.

There are no checklists or tools available to support costing dance programmes and minimal knowledge of how to categorise resources to identify the mainstream cost of delivery

Discrete Choice Experiment (DCE) methods to elicit the relative preferences and choices of girls aged 11-12 years are untried and untested, but it is important to capture how girls value dance among other competing leisure activities using a robust and acceptable method.

What this study adds

Providing programme cost data for a full trial of the BGDP programme is feasible. Around two-fifths of resources are development and research costs, so resources used to develop,

For peer review only

prepare and deliver the programme should be categorised separately, so the cost of the mainstream programme can be estimated accurately in a full trial.

Discrete Choice Experiment is an acceptable method to elicit preferences of girls aged 11-12 years.

At this point in their lives dance is popular choice when offered within the context of other competing choices for spending leisure time predicts positive recruitment and retention rates in after-school dance activities.

Participation in dance has opportunity costs for participants and parents extending beyond the funder that suggest a social model of cost should be considered for a full trial.

Robust evidence for the cost-effectiveness of physical activity complex interventions is important for knowing where to invest scarce resources and commission programmes to maximise health outcomes in primary prevention.[27 28 29] However, gathering robust evidence to support investment in public health interventions is a challenge.[30 31] Significant barriers remain and there is currently little guidance in how to conduct economic evaluation where behaviour change is associated with health outcomes determined beyond genetic inheritance by family, social and physical environments. [32 33]

Indicative programme cost data from the pilot economic evaluation indicated a substantial proportion of the intervention programme costs 42% occur at Stage 0 – the pre-programme planning and development stage. This is an important finding because it suggests provided BGDP is effective and cost-effective in a full trial, it would be substantially less costly to roll out in its mainstream form. All complex interventions in primary prevention are likely to generate a high proportion of upfront development costs that will not reoccur once a

For peer review only

programme is mainstreamed – an aspect of investment in public health interventions often overlooked by decision makers.

Application of DCE is an established technique in adult populations, but to our knowledge has not been applied previously in populations of children aged 11-12 years to establish values for the attributes of physical activities. This study has demonstrated application of DCE methods is feasible and acceptable to girls of this age. This is important because it suggests DCE could be applied in other studies with children to understand the concept of 'value' of an activity which plays an important role in recruitment, participation and maintenance which are all linked to intervention outcome. In addition to its acceptability the DCE method produced more complete data than the direct survey method in eliciting preference ranks for after-school leisure activities. These findings support a previous contention that DCE techniques may have merit over more 'traditional' survey methods [34] in eliciting preferences. However, more evidence would be required to fully support this finding.

Taken together, findings of the DCE and survey of leisure activity preference in this study indicate dance is a valued leisure activity among competing alternatives and reveals more about the attributes of dance classes in girls of this age that can be taken forward to maximise recruitment and retention in the BGDP programme. The findings of this study suggest that dance has immediate appeal as an after-school leisure activity among a range of strongly competing alternatives in girls of this age compared with older adolescents.[35] Girls in this study have a first rank preference for the attribute 'time remaining for other leisure activities on dance class days', over the 'cost of' and 'frequency of dance classes per week'. The finding that in the intervention group two hours is preferred to three hours remaining for other leisure activities on dance class days is significant. Overall these findings could suggest that at this point in their lives dance is popular choice in girls of this age when offered within

For peer review only

the context of other competing choices for spending leisure time. These are important findings because they predict positive recruitment rates and participation of girls aged 11-12 years in dance as a physical leisure time activity and in a full trial. [36]

Delivery of after-school dance classes is dependent upon substantial commitment from the girls giving up their after-school leisure time to participate in dancing. In turn, participation is dependent upon the willingness of parents and carers to support attendance and to provide encouragement and a means of travelling back home after school hours when school buses are not available. This pilot study suggests development of a social model of costing that reflects the cost of participants' and parents' time and opportunity costs are substantial elements of the intervention cost that could be captured if practical in a full trial.

However, methods and tools to capture 'hidden' cost items that facilitate the success of the intervention, but are not incurred by funders are not yet fully established. [17] How identification of costs falling outside the public sector that are relevant to programme implementation can be captured at a full trial stage needs to be considered carefully.[37] In a full trial resources used should be captured prospectively [38] and this pilot study has established categories of resource use are also important to consider to establish accurate mainstream programme costs.

CONCLUSIONS

The feasibility of providing costing data for full trial of the BGDP programme in is established and an embryonic resource use checklist has been developed. Resources used to develop and run the BGDP programme should be categorised separately in order for the mainstream delivery cost of BGDP to be estimated accurately in a full trial. A social model of costing that reflects participants and parents opportunity costs should be considered. BGDP

For peer review only

after-school dance classes have potential for sustained participation and cost-effective delivery, but a full trial using methodological learning from this study is required.

Competing interests

There are no competing interests.

Authors' Contributions

The quantitative analysis plan was conceived by JP, FC, RJ and SJS. Analyses were performed by JP and FC and interpretation by JP, FC, RJ, SJS and AMH. JP drafted the first version of the manuscript with additional sections provided by FC. All authors provided critical edits and revisions to the paper and have reviewed and approved the final version of the paper.

Data sharing statement No additional data are available

Funding

This research was funded by the National Prevention Research Initiative (http://npri.org.uk), consisting of the following funding partners: Alzheimer's Research Trust; Alzheimer's Society; Biotechnology and Biological Sciences Research Council, British Heart Foundation; Cancer Research UK; Chief Scientist Office; Scottish Government Health Directorate; Department of Health; Diabetes UK; Economic and Social Research Council; Engineering and Physical Sciences Research Council; Health & Social Care Research & Development Office for Northern Ireland; Medical Research Council; The Stroke Association; Welsh Assembly Government and World Cancer Research Fund [grant number G090084].

For peer review only

Acknowledgments

We would like to thank all the participants, dance teachers, school teachers and schools who participated in or assisted with this research. Professor Powell acknowledges discussion of and feedback from a draft version of this paper presented at the Health Economists Bristol (HEB) journal club http://www.bris.ac.uk/social-community-medicine/centres/healthecon in November 2011. Laura Davis project manager BGDP and Jade McNeil research associate are acknowledged for data collection leading to this paper. Fran Carroll's contemporaneous PhD research was supervised by Dr Alan A Montgomery at the time of DCE design and data analysis. Dr Jago is supported by a National Institute for Health Research career development fellowship. This work was also supported by DECIPHer, a UKCRC Public Health Research: Centre of Excellence. Funding from the specific funders under the auspices of the UK Clinical Research Collaboration is gratefully acknowledged. The other co-applicants of the main BGDP study, Professor Ashley R Cooper and Dr Alan A Montgomery are acknowledged. The views expressed in this publication are those of the authors and not necessarily those of the NHS, the National Institute for Health Research or the Department of Health.

For peer review only

References

1. Foresight. *Tackling obesities: future choices.* London: Government Office for Science, 2007.

2. Stamatakis E, Zaninotto P, Falaschetti E, Mindell, J. Head J. Time trends in childhood and adolescent obesity in England from 1995 to 2007 and projections of prevalence to 2015. *J Epidemiol Community Health* 2010; **64**:167-174.

3. Gunnell D, Frankel S, Nanchahal K, et al Childhood obesity and adult cardiovascular mortality: a 57 year follow-up study based on the Boyd Orr cohort. *Am J Clin Nutr* 1998: 67:1111-18.

4. Must A, Jacques PF, Dallal GE, et al. Long term morbidity and mortality of overweight adolescents – a follow-up of the Havard growth study of 1922 to 1935. *N Engl J Med* 1992: 327:1350-5.

5. Engeland A, Bjorge T, Sogaard AJ, et al. Body mass index in adolescence in relation to total mortality: 32-year Follow-up of 227,000 Norweigan boys and girls. *Am J Epidemiol* 2003:157:517-23.

6. Lancaster GA, Dodd S, Williamson P. Design and analysis of pilot studies: recommendations for good practice. *J Eval Clin Pract* 2004, 10, 2:307-312.

For peer review only

7. Craig P, Dieppe P, Macintyre S, Michie S, Nazareth I, Petticrew M. Developing and evaluating complex interventions: the new Medical Research Council guidance. *BMJ* 2008, 337:a1655.

8. Wolfenstetter SB. Conceptual framework for standard economic evaluation of physical activity programmes in primary prevention. *Prev Sci.*2011; 12(4):435-51.

9. Sheill, A. Hawe, P. Gold, L. Complex interventions or complex systems? Implications for health economic evaluation. *BMJ*, 2008; 336:1281-1283.

10. Weatherly H, Drummond M, Claxton K, et al. *Assessing the challenges of applying standard methods of economic evaluation to public health interventions.* York: Centre for Health Economics, 2009.

11. Anderson R. Systematic reviews of economic evaluations: utility or futility. *Health Econ*, 2010;19:350-364.

12. Jago R. Sebire S, Cooper AR, et al. Bristol Girls Dance Project Feasibility Trial: Outcome and process evaluation results. *Int J Behav Nutr Phys Act*, 2012; 9, 83.

13. Gorely T, Marshall S, Biddle SJH, Cameron N. The prevalence of leisure time sedentary behaviour and physical activity in adolescent girls: An ecological momentary assessment approach. *Int J Pediatr Obes*, 2007; 2:227-234.

14. Cheraghi-Sohi S, Bower P, Mead N, McDonald R, Whalley D, Roland M. Making sense of patient priorities: applying discrete choice methods in primary care using 'think aloud' technique. *Fam Pract*, 2007; 24: 276-282.

For peer review only

15. Green C, Gerard K. Exploring the social value of health-care interventions: a stated preference discrete choice experiment. *Health Econ*, 2009; 18:951-976.

16. Petrou S, McIntosh E. (2009) Women's Preferences for Attributes of First-Trimester Miscarriage Management: A Stated Preference Discrete-Choice Experiment. *Value Health*, 12,4:551-559.

17. Hollingworth W, Cohen D, Hawkins J, et al. Reducing smoking in adolescents: cost-effectiveness results from the cluster randomised ASSIST (A Stop Smoking in Schools Trial). *Nicotine & Tob Res*, 2011; 14, 2:161-168.

18. Jago, R. Davis, L. McNeil, J. et al. Adolescent girls' and parents' views on recruiting and retaining girls into an after-school dance intervention: implications for extra-curricular physical activity provision. *Int J Behav Nutr Phys Act*, 2011, 8, 91.

19. Bech,M. Gyrd-Hansen,D.. Effects coding in discrete choice experiments. *Health Econ*, 2005; 14,10.

20. STATACORP LP (2009) *STATA user's guide release 11.* Texas, USA: STATACORP LP.

21. Safalra [online] 2012. *Historical UK Inflation and price conversion rates.* Accessed on May 3rd 2012 at:- http://safalra.com/other/historical-uk-inflation-price-conversion/.

22. Oanda [online] 2012. *Historical exchange rates.* Accessed on May 3rd 2012 at:- http://www.oanda.com/currency/historical-rates/.

For peer review only

23. Fordham R, Barton GP. *Promotion of physical activity in children programme guidance: a cost-effectiveness scenario analysis.* London:NICE, 2008.

24. NICE public health guidance 17. *Promoting physical activity, active play and sport for pre-school and school-age children and young people in family, pre-school, school and community settings.* NICE:London, 2009.

25. Hampshire Dance and Laban (2007)[online] Accessed on December 7th 2012. *NRG Youth Dance and Health Report 2005-6.* http://www.hampshiredance.org.uk/documents/NRGEvaluationReport.pdf

26. TES [online] 2011 *Teacher's pay scales from September 2011 unchanged from 2010.* http://www.tes.co.uk/article.aspx?storycode=6000186

27. Rush B, Sheill A, Hawe P. A census of economic evaluations in health promotion. *Health Educ Res,* 2004; 19:707-719.

28. Kelly MP, McDaid D, Ludbrook A, Powell JE. *Economic appraisal of public health interventions.* London:Health Development Agency, 2005.

29. Anderson R. New MRC guidance on evaluating complex interventions. *BMJ,* 2008;337:944-5.

30. Buchanan J, Wolstenholme J, Foster C. *A rapid review of economic literature related to the promotion of physical activity, play and sport for pre-school and school-age children in family, pre-school, school and community settings.* London:NICE, 2008.

For peer review only

31. Richardson AK. Investing in public health: barriers and possible solutions. *J Public Health* 2012; 34:322-7.

32. Ogilvie D, Bull F, Powell J, et al. An applied ecological framework for evaluating infrastructure to promote walking and cycling: the iConnect study. *Am J Public Health*. 2011;101:473-481.

33. Powell J. Compression of morbidity outcomes key to investment in public health. *J Public Health* 2012; 34:329.

34. Alvarez-Farizo B, Hanley N, Barberan R. The value of leisure time: a contingent rating approach, *J Environ Plan Man*, 2001; 44(5):681-699.

35. O'Neill, J. Pate, R. Hooker, SP. The contribution of dance to daily physical activity among adolescent girls. *Int J Behav Nutr Phys Act*, 2011; 8:87.

36. Nader PR, Bradley RH, Houts RM, McRitchie SL, O'Brien M: Moderate-to-vigorous physical activity from ages 9 to 15 years. *JAMA* 2008, 300:295-305.

37. Alvarez-Farizo B, Hanley N, Barberan R. The value of leisure time: a contingent rating approach, *J Environ Plan Man*, 2001; 44,5:681-699.

38. Ridyard C, Hughes D. Methods for the collection of resource use data within clinical trials: a systematic review of the studies funded by the UK Health Technology Assessment Programme. *Value Health*, 2010; 13, 8:867-872.

For peer review only

TABLES

Table I. Attributes and levels of the Discrete Choice Experiment and the four choice sets given to participants

Level of attributes	Attributes		
	Frequency of dance classes per week	Cost per session	Hours left for other leisure activities on that day
Upper	2 dance classes per week	£1 per session	Leaving 3 hours for other leisure activities on the evening of the dance session
Lower	1 dance class per week	50p per session	Leaving 2 hours for other leisure activities on the evening of the dance session

1A<br>You take one after school dance class each week at a cost of £1 per class leaving you 3 hours on that evening for other leisure activities	1B<br>You take two after school dance classes each week at a cost of 50p per class leaving you 2 hours on those evenings for other leisure activities
2A<br>You take two after school dance classes each week at a cost of £1 per class leaving you 2 hours on those evenings for other leisure activities	2B<br>You take one after school dance class each week at a cost of 50p per class leaving you 3 hours on that evening for other leisure activities
3A<br>You take one after school dance class each week at a cost of 50p per class leaving you 2 hours on that evening for other leisure activities	3B<br>You take two after school dance classes each week at a cost of £1 per class leaving you 3 hours on those evenings for other leisure activities
4A<br>You take two after school dance classes each week at a cost of 50p per class leaving you 3 hours on those evenings for other leisure activities	4B<br>You take one after school dance class each week at a cost of £1 per class leaving you 2 hours on that evening for other leisure activities

Table II. Resources, estimated total costs and average cost per participant 2010-11 prices

Stage of BGDP	*Description of resources used*	*Total cost $ (£, €) 2010-11 prices*
**Stage 0** Pre-Programme planning development	Nine hours to adapt training programme for hip hop genre, two 3 hour sessions to prepare dance teachers for 9 week intervention delivery by lead dance teacher, 20 hours preparation by 3 dance teachers, disclosures CRB forms	**$8,525** £5,326 €6,238
**Stage 1** Programme preparation	Space hire for two 3 hour dance taster sessions, dance teacher's delivery of two 3 hour taster sessions, 2 control recruitment presentations (90 minutes), postage costs	**$936** £585 €685
**Stage 2** Programme delivery	Delivery of 18 dance classes at 90 minutes per class over 9 weeks in three schools, incentives for intervention schools, incentives for control schools data collection, refreshments	**$9,575** £5,982 €7,007
**Stage 3** Programme reinforcement	Three dance workshops for two control schools at 90 minutes per workshop, three 6 hour end of intervention performance events for parents	**$1,153** £720 €843
BGDP Stages 0-3	**Total cost**	**$20,189** £12,613 €14,773
BGDP Stages 1-3	**Total cost**	**$11,664** £7,287 €8,535
BGDP Stages 1-3	**Total cost minus research costs**	**$7,666** £4,789 €5,609
	**BGDP Cost per participant (N = 90)**	**$85.17** £53.21 €62.32

For peer review only

Table III. Preference rankings of first choice leisure activities at each time point N (%)

	Time 2		Time 1		Baseline Time 0	
**After-school leisure activity**	*Ranking*	*N (%)*	*Ranking*	*N (%)*	*Ranking*	*N (%)*
Go around with friends to shopping centres, streets, parks just for fun	1	46 (26)	1	33 (25)	2	12 (18)
Use the internet for fun: chats, YouTube, Facebook, Bebo, Myspace, looking for music (do not include school homework)	2	31 (17)	3	20 (15)	3	8 (12)
Take part in sports, athletics or physical activity	2	31 (17)	2	22 (17)	1	13 (20)
Play with or see friends at your home or their homes	3	21 (12)	4	11 (9)	5	5 (7)
Read books for enjoyment (do not include school books)	4	13 (7)	5	10 (8)	4	6 (9)
Go to discos or dance classes	5	11 (6)	8	5 (4)	5	5 (7)
Play a musical instrument, sing, draw, paint or write	6	9 (5)	4	11 (9)	3	8 (12)
Send text messages or use Twitter on your mobile phone	7	8 (5)	7	6 (5)	5	5 (7)
Play computer games	8	4 (2)	6	8 (6)	6	4 (6)
Watch TV DVDs or playbacks of programmes	8	4 (2)	9	4 (3)	7	2 (3)
Total of valid* responses/total responses		178/210		130/210		68/210

*A valid response = each after-school leisure activity is ranked by a separate number between 1 and 10 by individual participant

For peer review only

Table IV. Order of preferences and preference proportions of levels indicating the value of dance classes at t0 (week 0) and t1 (week 9) by group allocation

	Level preferences *Preference proportions*[1]			
**Ranking of Attributes**	***Control time 0 (n=104/120*)***	***Control time 1 (n=104/120*)***	***Intervention time 0 (n=80/90*)***	***Intervention time 1 (n=80/90*)***
**1. Time for other activities on dance class days** *2 hrs/day v 3hrs /day*	Having 2 hours remaining for other leisure activities on dance class days *67% v 33%*	Having 3 hours remaining for other leisure activities on dance class days *35% v 65%*	Having 2 hours remaining for other leisure activities on dance class days *82% v 18%*	Having 2 hours remaining for other leisure activities on dance class days *68% v 32%*
**2.Cost** *50p v £1*	£1 cost *39% v 61%*	50p cost *58% v 42%*	£1 cost *28% v 72%*	£1 cost *37% v 63%*
**3. Frequency of dance classes per week** *1 p/wk v 2 p/wk*	2 classes per week *41% v 59%*	2 classes per week *36% v 64%*	2 classes per week *38% v 62%*	2 classes per week *44% v 56%*

[1]Preference proportions holding all else equal, the proportion of the sample that would choose particular level over the other within that attribute
**Number of valid responses from total possible responses*

For peer review only

Bristol Girls Dance Project feasibility study: using a pilot economic evaluation to inform design of a full trial

Journal:	*BMJ Open*
Manuscript ID:	bmjopen-2013-003726.R1
Article Type:	Research
Date Submitted by the Author:	22-Oct-2013

Complete List of Authors:	Powell, Jane; University of the West of England, Bristol, Health and Social Sciences<br>Carroll, Fran; University of Bristol, School of Social and Community Medicine<br>Sebire, Simon; University of Bristol, Centre for Exercise Nutrition and Health Sciences<br>Haase, Anne; University of Bristol, Centre for Exercise Nutrition and Health Sciences<br>Jago, Russ; University of Bristol, Centre for Exercise, Nutrition and Health Sciences

**Primary Subject Heading**:	Public health
Secondary Subject Heading:	Health economics, Sports and exercise medicine
Keywords:	SPORTS MEDICINE, HEALTH ECONOMICS, PUBLIC HEALTH

SCHOLARONE™
Manuscripts

For peer review only

RESEARCH PILOT STUDY

Bristol Girls Dance Project feasibility study: using a pilot economic evaluation to inform design of a full trial

Powell, J.[1§] Carroll, FE.[2] Sebire SJ.[3] Haase, AM.[3] Jago, R.[3]

[1] Professor of Public Health Economics, Department of Health and Social Sciences, University of the West of England, Glenside Campus, Stapleton, Bristol, BS16 1DD, UK.

[2]Dr Fran E Carroll, Research Assistant, School of Social and Community Medicine, University of Bristol, BS8 2PS, UK.

[3]Dr Simon J Sebire, Post-Doctoral Researcher, Centre for Exercise Nutrition and Health Sciences, School for Policy Studies, University of Bristol, BS8 1TZ, UK.

[3]Dr Anne M Haase, Senior Lecturer in Exercise, Nutrition & Health, Centre for Exercise, Nutrition and Health Sciences, School for Policy Studies, University of Bristol, BS8 1TZ, UK.

[3]Professor Russell Jago, Professor of Paediatric Physical Activity & Public Health Exercise, Nutrition & Health Sciences, Centre for Exercise, Nutrition & Health Sciences, School for Policy Studies, University of Bristol, BS8 1TZ, UK.

[§]Corresponding author Professor Jane Powell, Department of Health and Social Sciences, University of the West of England, Bristol, BS16 1DD, UK. 0044 117 3288752

For peer review only

ABSTRACT

Wider context: There is currently little guidance for pilot trial economic evaluation where health outcomes and costs are influenced by a range of wider determinants and factors.

Objectives: This paper presents the findings of a pilot economic evaluation study running alongside the Bristol Girls Dance Project (BGDP) feasibility study.

Design: Three-arm, cluster randomised, controlled pilot trial and economic evaluation. Seven schools (n=210) from the Bristol and greater Bristol area, UK were randomly allocated to the intervention arm 3 schools (n= 90) and the control arms 4 schools (n=120).

Intervention: Girls aged 11-12 years with parental consent were provided with two, 90-minute dance sessions per week for 9-weeks at school facilities.

Economic outcome measures: Programme costs and girls' preferences for attributes of dance and preferences for competing leisure time activities were measured.

Results: The mainstream average cost of the BDGP programme (not including research, control and dance teacher training costs) per school was $2,126.40; £1,329; €1,555 and per participant $70.90; £44.31; €51.84 in 2010-11 prices. Discrete Choice Experiment (DCE) methods are acceptable to girls of this age indicating time available for other leisure activities on dance class days is the attribute girls valued most and 2 hours leisure time remaining preferred to 3 hours.

Conclusions: This pilot study indicates providing full cost data for a future trial of the BGDP programme is feasible and practical. There is no evidence from preference data to support adjustment to intervention design. A future economic evaluation is likely to be successful utilising the resource use checklist developed. The importance of categorising separately

For peer review only

resources used to develop, prepare, deliver and maintain the programme to estimate mainstream costs accurately is demonstrated.

Strengths and limitations of this study

This pilot study used a systematic approach where there is currently minimal evidence to determine the costs of implementing a pilot dance intervention in girls 11-12 years old. The study has produced findings about girls' preferences for dance and an embryonic costing tool that can be applied to design and conduct an economic evaluation alongside a full cluster RCT. This feasibility and exploratory pilot study is powered to test the intervention concept, the feasibility of obtaining programme cost data in categories and the evidence required to power a full cluster RCT in the future. Consequently, the variation in programme costs at the school level has not been captured and this is a limitation of the cost estimates presented.

BACKGROUND

Recent influential studies attach substantial economic and social costs to obesity prevalence projections.[1 2] These forecasts are based upon a body of research from long term cohort studies which suggest that change in the prevalence of obesity in children and adolescents born at the millennium is likely to lead to increased health risks in middle-age irrespective of adult adiposity.[3 4 5] Consequently, there is a need for new interventions that focus on preventing obesity or changing diet or physical activity; the two behaviours that are central to the accrual of body mass.

As well as being a health and wellbeing issue, children's obesity also has serious economic impacts. Scarce resources with competing uses in all health systems and the need to decide

For peer review only

between new, ‘efficacious’ primary prevention physical activity programme interventions on the grounds of cost-effectiveness, has increased the significance of economic evaluation as a concept and methodology. Recent guidance from the UK Medical Research Council (MRC) for the development and evaluation of complex behavioural interventions suggests that efficacy and cost-effectiveness should be established before programmes are implemented at the population level.[6 7] However, the meaningful determination of these criteria is often problematic in primary prevention and guidelines for the design and conduct of economic evaluation of complex interventions are at an early stage of development.[8 9 10 11] It is therefore important to develop the conceptual and measurement process by which effectiveness and cost-effectiveness of complex physical activity interventions can be evaluated in a full trial using a pilot study.

The main findings of the Bristol Girls Dance Project (BGDP) feasibility trial concerning process evaluation, outcomes and effectiveness have been published elsewhere .[12] This part of the study suggested that it is feasible to deliver the intervention and that participating in dance has the potential to yield change in moderate to vigorous physical activity (MVPA) among 11-12 year old girls (school year 7) , but a larger randomised controlled trial (RCT) would be needed to fully evaluate its effectiveness and cost-effectiveness. [12] In the absence of robust evidence for the cost and outcome of dance interventions, other aims of the feasibility pilot were to refine the information required to sufficiently power a full trial and to use the preference data to inform potential refinements to intervention design.

Preferences for competing after-school activities are potential determinants of the economic benefit of dance intervention, as increased physical activity must be valued in order for it to be maintained [8] and to have potential for long-term impact on physical activity levels. In this study, Discrete Choice Experiment (DCE) and survey methods are applied to examine two separate, but complementary aspects of value –preferences for the attributes of dance classes

For peer review only

and preferences for dance among other competing alternatives for spending leisure time respectively. Physical activity levels decline during youth [13] with the start of secondary school being a critical period of change, so it was important to establish comparative preferences for after-school, leisure activities on weekdays.

Value is a concept germane to recruitment and retention rates and linked to the outcome dimension of the BGDP intervention and therefore important to examine in detail. DCE works on the premise that any 'product', for example a healthcare treatment or physical activity programme, can be described by its characteristics, or attributes, and the extent to which an individual values a 'product' is dependent on the level of these characteristics. [14 15 16]

Thus, this paper reports the findings of a pilot economic evaluation of the Bristol Girls Dance Project for girls aged 11-12 years in a primary school setting in England that can be applied to design and conduct a future full trial and economic evaluation.

ARTICLE SUMMARY

Article focus

To examine whether the proposed methods for collection of resource use data for estimating the costs are feasible, practical and likely to be successful in full trial economic evaluation.

To understand preferences for after-school dance classes in the context of weekday leisure time at age 11-12 years that can be applied to inform intervention design.

For peer review only

Key messages

Time available for other leisure activities on dance class days is an attribute of after school dance classes girls aged 10-11 years valued most - followed by cost and frequency of dance classes per week.

2 hours available for other leisure activities on dance class days was preferred to 3 hours suggesting after-school dance classes are valued compared with other ways to spend leisure time after school on weekdays

Resources used in the development, preparation, delivery and maintenance of dance classes should be categorised separately in stages in order to identify the mainstream cost of the programme intervention to commissioners

METHODS

Bristol Girls Dance Project (BGDP) feasibility study

BGDP was a three-arm, parallel group, cluster randomised, controlled pilot trial with schools as the unit of allocation. Seven schools from three school districts, Bristol, Bath and South Gloucestershire (UK) were recruited to take part in the study from schools in these districts with no current after school dance provision.[12] The hip-hop and street dance style of dance to popular music was facilitated by a professional dance teacher.

Stratifying by school district, three schools were randomly allocated to the intervention arm (n= 90) and four schools to the two control arms (n=120) and each school was assigned a dance teacher to lead the sessions. Randomisation was conducted by an independent member of the clinical trials unit at Bristol University using computer-generated random sequences and codes for school district and school name. The three intervention schools received two,

For peer review only

90-minute after-school dance classes per week for 9 weeks selected to allow the entire programme to be delivered within a school term. Pilot work had suggested that dance is a very attractive form of PA for girls, so the control element was designed to ascertain whether offering a dance workshop at the end of the research process (i.e., after the last data collection), would affect either retention or the quality of data provided by participants. We therefore utilized a three-arm design with two different control groups. In two schools, participants were provided with small thank you gifts for each wave of data collection. In the other two control schools participants were provided with the same small thank you gifts, as well as a half-day dance workshop at the end of the study.

Sample size

This feasibility study was powered to test the intervention concept and to provide the necessary information to calculate the sample size of a full cluster RCT and economic evaluation of an after school dance programme. The important parameters detection of 10 minutes difference in MVPA per weekday (50 minutes per week) between the intervention and control groups, intra-class correlation for weekday MVPA at time 2 and associated confidence intervals have been reported and profiled in another paper from this study. [12] For practical reasons the sample was limited to thirty girls aged 11-12 years per school. Girls were recruited from each school at random from those with parental consent.

For peer review only

Economic measures

1) *Discrete Choice Experiment (DCE) and survey of preference ranking and use of leisure time*

BGDP formative qualitative work indicated the frequency of after-school dance classes per week, cost per session and how much leisure time is left over on dance class days for other leisure activities are important considerations for girls in deciding whether to participate.[17] Participants were asked to select the 'dance class scenario' they preferred from a pair of options. Table I presents the 4 paired scenarios (1A:1B, 2A:2B, 3A:3B, 4A:4B) consisting of a randomly determined combination of three attributes, each with two levels.

Table I

Four paired choice scenarios were administered to 210 girls in seven schools - 3 intervention schools (n=90) and 4 control schools (n=120). Measures were taken at baseline (time 0) and at 9 weeks (time 1) using large cards, and girls' preferred choice for each pair of scenarios was recorded by the project team. Two time points were needed to establish change in preferences before and after the intervention. Participants were also asked to give preference ratings for ten leisure activities on weekdays by survey using a ten-point scale (1 = favourite; 10 = least favourite). Participant responses were collected on Personal Digital Assistants (PDAs) and downloaded to a customised database.

2) *Resource use-cost*

At commencement of this pilot study there was minimal evidence on which to draw in identifying costs that might be included in a resource use checklist. One report from the National Institute for Health and Clinical Excellence (NICE) had modelled the cost of

For peer review only

delivering dance classes to young children and produced some ball park cost estimates. [18 19] These were based on an account of the resources used in delivery of a dance programme for girls by Hampshire Dance and Laban [20] in which resources had been identified, but not costed. These uncontrolled studies provided a starting point and an opportunity to produce more complete and accurate costing data from the Bristol Girls Dance Project feasibility pilot trial in which the volume of resources used and prices could be treated separately. The cost items identified by NICE were entered into a database and data collected using time-sheets and expense sheets were collected by the project team. These cost estimates and some estimates for teacher time to manage behaviour derived by the first author of this paper are detailed in Table II.[18 19 20] Table II was used as a template for identifying and costing resources in the BGDP feasibility study.

TABLE II here

Ethics

Potential participants in all seven schools were told that there was a maximum of 30 randomly assigned spaces at the dance classes. The study was approved by a University of Bristol ethics committee and informed parental consent was obtained for all participants.

Analyses

Proportions of the sample ranking ten weekday leisure activities as first choice activity (rank = 1) were calculated after participants had rated all ten leisure activities from 1 to 10. Responses from participants with repetition of ratings for one or more leisure activities or missing ratings for leisure activities were excluded. Overall, where the proportion of the sample rating activities as their first choice was the same, these activities were assigned the

For peer review only

same rank across all ten activities. DCE data was 'effects-coded' [21] using STATA [22] and analysed using conditional logistic regression. Effects coding is similar to dummy variable coding, but is preferable in this instance because interaction or trade-off between the attributes is likely to take place as well as a main effect. The coefficients for each attribute are a measure of the influence of that attribute level on choice. Positive values represent a positive influence on choice, or in other words, a preference for that level of an attribute. These results can be used to establish girls' overall preferences for attributes, as well as the order of their preferences (i.e. which attribute is most and least important). Participants with missing data were excluded from the DCE analysis.

Total and average cost estimates from a funder perspective were identified and derived for BGDP based on staged timing, quantity, frequency and price of resource use in 2010-11 prices. Expenses including travel, intervention programme entry incentives, postage, Criminal Records Bureau (CRB) applications were accessed from the database kept by the project team. Girls in the control schools received small thank you gifts at each data collection they attended. Space hire did not incur costs, but estimates of the cost of space hire for dance class delivery are included because they are costs connected with alternative use of space in schools. School overhead and capital costs are not included.

Grouping costs to enable estimation of the mainstream cost adopted the categories used in (A Stop Smoking in Schools Trial) ASSIST.[23] Stage 0 intervention planning, development and training costs, stage 1 intervention preparation, stage 2 intervention delivery and stage 3 intervention maintenance costs were separately identified. Training costs for dance teachers are identified separately. Costs associated with running the research study, control group incentives for data collection, control school dance workshops and recruitment events) would

For peer review only

not recur during mainstream implementation, but these costs are included for clarity and completeness. All costs connected with tasks undertaken by the research team are not included.

RESULTS

Identification and timing of resources used

Table III identifies and describes at four stages the resources use of the BGDP programme and presents total cost estimates. The proportion of total costs incurred were 41% at stage 0, 7% at stage 1, 46% at stage 2 and 6% at stage 3. At stage 0 half of the costs are dance teacher preparation and training time which arguably would be incurred in part in delivery of a mainstream form of the programme. Eighteen BGDP dance classes (2 classes per week for 9 weeks) of 90 minutes duration were delivered to 90 girls in three intervention schools (30 per school) for 81 hours (27 hours per school) at a total estimated cost $6,380, £3, 988, €4,666 in 2010-11 prices.[24 25] The average cost of the BDGP programme in its mainstream form per school was $2,126.40; £1,329; €1,555 and per participant $70.90; £44.31; €51.84 in 2010-11 prices. If training costs for dance teachers on the BGDP were included to the mainstream cost this would add $1280; £800; €928 to the cost per school and $43; £27; €31.60 to the cost per pupil. These are not insubstantial additions, but are at the high end of training costs because this new dance programme was properly prepared for delivery. Training costs for the delivery of an established dance programme are likely to be lower. It was not possible to calculate confidence intervals for average programme costs per school or per participant (see limitations of this pilot study).

Table III here

For peer review only

The shorter and less intensive NRG programme costed in the NICE report [19] comprised 10 dance classes of 60 minutes duration for 24 girls (an assumed number) in 14 groups or schools (n=336 girls) for 140 hours with a cost per participant of $98.14; £61.31; €71.81, in 2010-11 prices. This includes 140 hours of teacher time sourced from national pay scales for England at £23.57 per hour at 2010-11 prices.[26]

Preferences for leisure activities

All girls were asked to rank ten after-school leisure activities by first preference activity. Table III presents proportions across the participants (n=210) for preference ranks for after-school leisure activities for all group allocations at each time point demonstrating consistency in preference ranks indicating girls' selection of first choice leisure activities at each time point. The after-school leisure activities indicating the highest proportion of first choice preference rankings at each time point include 'hanging out with friends away from home just for fun' (Ranking at t2=1, t1=1, t0=2); 'take part in sports, athletics or physical activity' (Ranking at t2=2, t1=2, t0=1) and 'using the internet for fun: chats, YouTube, Facebook, Bebo, Myspace, looking for music' (Ranking at t0=3, t1=3, t2=2). Valid responses were included in the analyses. Valid responses as a proportion of total responses for the survey ranking leisure activities were t2=178/210, t1=130/210, t0=68/210 across all group allocations indicating particularly at baseline the participants experienced some problems using a hand held PDA to rank and rate the weekday after-school leisure activities.

Table IV here

DCE results

For peer review only

The p values for the regression coefficients in Table IV indicate girls in this sample have a preference for “time left for other leisure activities on dance class days”, over the “cost of” and “frequency of dance classes per week”. Analysis of preference levels within each attribute suggests 2 hours is preferred to 3 hours remaining for other leisure activities on dance class days. This pattern was consistent in all intervention and control groups at t0 and t1. Girls were least concerned with the frequency of dance classes per week with preference proportions suggesting 2 classes were preferred to 1 dance class per week in both intervention groups and the baseline control group.

Table V here

DISCUSSION

What is already known on this topic

There is minimal guidance to support how economic evaluations of complex public health interventions should be designed and conducted in school and community settings.

There are no checklists or tools available to support costing dance programmes and minimal knowledge of how to categorise resources to identify the mainstream cost of delivery.

Discrete Choice Experiment (DCE) methods to elicit the relative preferences and choices of girls aged 11-12 years are untried and untested, but it is important to capture how girls value dance among other competing leisure activities using a robust and acceptable method.

What this study adds

For peer review only

Providing programme cost data for a full trial of the BGDP programme is feasible, practical and likely to be successful. Around two-thirds of resources are development and research control costs, so resources used to develop, prepare and deliver these programmes should be categorised separately, so the cost of the mainstream programme can be estimated accurately.

Discrete Choice Experiment is an acceptable method to elicit preferences of girls aged 11-12 years.

At this point in their lives after-school dance is an activity valued by girls when offered within the context of other competing choices and parental support for activities for spending leisure time after school on weekdays.

Participation in after-school dance classes has opportunity costs for participants and parents extending beyond the funder that suggest a social model of cost should be considered for to capture the costs associated with intervention outcome.

Robust evidence for the cost-effectiveness of physical activity complex interventions is important for knowing where to invest scarce resources and commission programmes to maximise health outcomes in primary prevention.[27 28 29] However, gathering robust evidence to support investment in public health interventions is a challenge.[30 31] Significant barriers remain and there is currently little guidance in how to conduct economic evaluation where behaviour change is associated with health outcomes determined beyond genetic inheritance by family, social and physical environments. [32 33]

Indicative programme cost data from the pilot economic evaluation indicated a substantial proportion of the intervention programme costs 41% occur at Stage 0 – the pre-programme development stage. This is an important finding because it suggests provided BGDP is effective and cost-effective in a full trial, it would be substantially less costly to roll out in its

For peer review only

mainstream form. All complex interventions in primary prevention are likely to generate a high proportion of upfront development costs that will not reoccur once a programme is mainstreamed – an aspect of investment in public health interventions often overlooked by decision makers.

Application of DCE is an established technique in adult populations, but to our knowledge has not been applied previously in populations of children aged 11-12 years to establish values for the attributes of physical activities. This study has demonstrated application of DCE methods is feasible and acceptable to girls of this age. This is important because it suggests DCE could be applied in other studies with children to understand the concept of 'value' of an activity which plays an important role in recruitment, participation and maintenance of participants which are all linked to intervention outcome. In addition to its acceptability in this study the DCE method has produced more complete and valid data than the direct survey method in eliciting preference ranks for after-school leisure activities. These findings support a previous contention that DCE techniques may have merit over more 'traditional' survey methods [34] in eliciting preferences. However, more evidence would be required to fully support this finding.

Taken together, findings of the DCE and survey of leisure activity preference in this study indicate dance is a valued leisure activity among competing alternatives and reveals more about the attributes of dance classes in girls of this age that can be taken forward to maximise recruitment and retention in the BGDP programme. The findings of this study suggest that dance has immediate appeal as an after-school leisure activity among a range of strongly competing alternatives in girls of this age compared with older adolescents.[35] Girls in this study have a first rank preference for the attribute 'time remaining for other leisure activities on dance class days', over the 'cost of' and 'frequency of dance classes per week'. The

For peer review only

finding that in the intervention group two hours is preferred to three hours remaining for other leisure activities on dance class days is significant. Overall these findings could suggest that at this point in their lives dance is valued by girls as a physical and social activity when offered within the context of competing and constrained choices for spending leisure time at this age. For example, at this age girls are not likely to be able to go to 'discos or dance classes' without parents or carers or to 'hang around on street corners with friends' and these issues may have affected their responses in the survey. These are important findings because they predict positive recruitment rates and participation of girls aged 11-12 years in dance as a physical leisure time activity and in a full trial. [36]

Delivery of after-school dance classes is dependent upon substantial commitment from the girls giving up their after-school leisure time to participate in dancing. In turn, participation is dependent upon the willingness of parents and carers to support attendance and to provide encouragement and a means of travelling back home after school hours when school buses are not available. This pilot study suggests development of a social model of costing that reflects the cost of participants' and parents' time and opportunity costs are substantial elements of the intervention cost that could be captured, if practical, in a full trial.

However, methods and tools to capture 'hidden' cost items that facilitate the success of the intervention, but are not incurred by funders are not yet fully established. [23] Where to include training costs in these metrics is a question that remains for a future trial as they should arguably be included in mainstream cost estimation despite their categorisation as development costs. How identification of costs falling outside the public sector that are relevant to programme implementation can be captured at a full trial stage also needs to be considered carefully.[37] In a full trial resources used should be captured prospectively [38] and this pilot study has established categories of resource use are also important to consider to establish accurate mainstream programme costs.

For peer review only

CONCLUSIONS

The feasibility of providing costing data for full trial of the BGDP programme in is established and an embryonic resource use checklist has been developed. Resources used to develop and run the BGDP programme should be categorised separately in order for the mainstream delivery cost of BGDP to be estimated accurately in a full trial. A social model of costing that reflects participants and parents opportunity costs should be considered. BGDP after-school dance classes have potential for sustained participation and cost-effective delivery, but a full trial using methodological learning from this study is required.

Competing interests

There are no competing interests.

Authors' Contributions

The quantitative analysis plan was conceived by JP, FC, RJ and SJS. Analyses were performed by JP and FC and interpretation by JP, FC, RJ, SJS and AMH. JP drafted the first version of the manuscript with additional sections provided by FC. All authors provided critical edits and revisions to the paper and have reviewed and approved the final version of the paper.

Data sharing statement No additional data are available

For peer review only

Funding

This research was funded by the National Prevention Research Initiative (http://npri.org.uk), consisting of the following funding partners: Alzheimer's Research Trust; Alzheimer's Society; Biotechnology and Biological Sciences Research Council, British Heart Foundation; Cancer Research UK; Chief Scientist Office; Scottish Government Health Directorate; Department of Health; Diabetes UK; Economic and Social Research Council; Engineering and Physical Sciences Research Council; Health & Social Care Research & Development Office for Northern Ireland; Medical Research Council; The Stroke Association; Welsh Assembly Government and World Cancer Research Fund [grant number G090084].

Acknowledgments

We would like to thank all the participants, dance teachers, school teachers and schools who participated in or assisted with this research. Professor Powell acknowledges discussion of and feedback from a draft version of this paper presented at the Health Economists Bristol (HEB) journal club http://www.bris.ac.uk/social-community-medicine/centres/healthecon in November 2011. Laura Davis project manager BGDP and Jade McNeil research associate are acknowledged for data collection leading to this paper. Fran Carroll's contemporaneous PhD research was supervised by Dr Alan A Montgomery at the time of DCE design and data analysis. Dr Jago is supported by a National Institute for Health Research career development fellowship. This work was also supported by DECIPHer, a UKCRC Public Health Research: Centre of Excellence. Funding from the specific funders under the auspices of the UK Clinical Research Collaboration is gratefully acknowledged. The other co-applicants of the main BGDP study, Professor Ashley R Cooper and Dr Alan A Montgomery are acknowledged. The views expressed in this publication are those of the authors and not

For peer review only

necessarily those of the NHS, the National Institute for Health Research or the Department of Health.

For peer review only

References

1. Foresight. *Tackling obesities: future choices.* London: Government Office for Science, 2007.

2. Stamatakis E, Zaninotto P, Falaschetti E, Mindell, J. Head J. Time trends in childhood and adolescent obesity in England from 1995 to 2007 and projections of prevalence to 2015. *J Epidemiol Community Health* 2010; **64**:167-174.

3. Gunnell D, Frankel S, Nanchahal K, et al Childhood obesity and adult cardiovascular mortality: a 57 year follow-up study based on the Boyd Orr cohort. *Am J Clin Nutr* 1998: 67:1111-18.

4. Must A, Jacques PF, Dallal GE, et al. Long term morbidity and mortality of overweight adolescents – a follow-up of the Havard growth study of 1922 to 1935. *N Engl J Med* 1992: 327:1350-5.

5. Engeland A, Bjorge T, Sogaard AJ, et al. Body mass index in adolescence in relation to total mortality: 32-year Follow-up of 227,000 Norweigan boys and girls. *Am J Epidemiol* 2003:157:517-23.

6. Lancaster GA, Dodd S, Williamson P. Design and analysis of pilot studies: recommendations for good practice. *J Eval Clin Pract* 2004, 10, 2:307-312.

For peer review only

7. Craig P, Dieppe P, Macintyre S, Michie S, Nazareth I, Petticrew M. Developing and evaluating complex interventions: the new Medical Research Council guidance. *BMJ* 2008, 337:a1655.

8. Wolfenstetter SB. Conceptual framework for standard economic evaluation of physical activity programmes in primary prevention. *Prev Sci.*2011; 12(4):435-51.

9. Sheill, A. Hawe, P. Gold, L. Complex interventions or complex systems? Implications for health economic evaluation. *BMJ*, 2008; 336:1281-1283.

10. Weatherly H, Drummond M, Claxton K, et al. *Assessing the challenges of applying standard methods of economic evaluation to public health interventions.* York: Centre for Health Economics, 2009.

11. Anderson R. Systematic reviews of economic evaluations: utility or futility. *Health Econ*, 2010;19:350-364.

12. Jago R. Sebire S, Cooper AR, et al. Bristol Girls Dance Project Feasibility Trial: Outcome and process evaluation results. *Int J Behav Nutr Phys Act*, 2012; 9, 83.

13. Gorely T, Marshall S, Biddle SJH, Cameron N. The prevalence of leisure time sedentary behaviour and physical activity in adolescent girls: An ecological momentary assessment approach. *Int J Pediatr Obes*, 2007; 2:227-234.

For peer review only

14. Cheraghi-Sohi S, Bower P, Mead N, McDonald R, Whalley D, Roland M. Making sense of patient priorities: applying discrete choice methods in primary care using 'think aloud' technique. *Fam Pract*, 2007; 24: 276-282.

15. Green C, Gerard K. Exploring the social value of health-care interventions: a stated preference discrete choice experiment. *Health Econ*, 2009; 18:951-976.

16. Petrou S, McIntosh E. (2009) Women's Preferences for Attributes of First-Trimester Miscarriage Management: A Stated Preference Discrete-Choice Experiment. *Value Health*, 12,4:551-559.

17. Jago, R. Davis, L. McNeil, J. et al. Adolescent girls' and parents' views on recruiting and retaining girls into an after-school dance intervention: implications for extra-curricular physical activity provision. *Int J Behav Nutr Phys Act*, 2011, 8, 91.

18. Fordham R, Barton GP. *Promotion of physical activity in children programme guidance: a cost-effectiveness scenario analysis.* London:NICE, 2008.

19. NICE public health guidance 17. *Promoting physical activity, active play and sport for pre-school and school-age children and young people in family, pre-school, school and community settings.* NICE:London, 2009.

20. Hampshire Dance and Laban (2007)[online] Accessed on December 7th 2012. *NRG Youth Dance and Health Report 2005-6.* http://www.hampshiredance.org.uk/documents/NRGEvaluationReport.pdf

For peer review only

21. Bech,M. Gyrd-Hansen,D.. Effects coding in discrete choice experiments. *Health Econ*, 2005; 14,10.

22. STATACORP LP (2009) *STATA user's guide release 11.* Texas, USA: STATACORP LP.

23. Hollingworth W, Cohen D, Hawkins J, et al. Reducing smoking in adolescents: cost-effectiveness results from the cluster randomised ASSIST (A Stop Smoking in Schools Trial). *Nicotine & Tob Res*, 2011; 14, 2:161-168.

24. Safalra [online] 2012. *Historical UK Inflation and price conversion rates.* Accessed on May 3rd 2012 at:- http://safalra.com/other/historical-uk-inflation-price-conversion/.

25. Oanda [online] 2012. *Historical exchange rates.* Accessed on May 3rd 2012 at:- http://www.oanda.com/currency/historical-rates/.

26. TES [online] 2011 *Teacher's pay scales from September 2011 unchanged from 2010.* http://www.tes.co.uk/article.aspx?storycode=6000186

27. Rush B, Sheill A, Hawe P. A census of economic evaluations in health promotion. *Health Educ Res,* 2004; 19:707-719.

28. Kelly MP, McDaid D, Ludbrook A, Powell JE. *Economic appraisal of public health interventions.* London:Health Development Agency, 2005.

29. Anderson R. New MRC guidance on evaluating complex interventions. *BMJ,* 2008;337:944-5.

For peer review only

30. Buchanan J, Wolstenholme J, Foster C. *A rapid review of economic literature related to the promotion of physical activity, play and sport for pre-school and school-age children in family, pre-school, school and community settings.* London:NICE, 2008.

31. Richardson AK. Investing in public health: barriers and possible solutions. *J Public Health* 2012; 34:322-7.

32. Ogilvie D, Bull F, Powell J, et al. An applied ecological framework for evaluating infrastructure to promote walking and cycling: the iConnect study. *Am J Public Health*. 2011;101:473-481.

33. Powell J. Compression of morbidity outcomes key to investment in public health. *J Public Health* 2012; 34:329.

34. Alvarez-Farizo B, Hanley N, Barberan R. The value of leisure time: a contingent rating approach, *J Environ Plan Man*, 2001; 44(5):681-699.

35. O'Neill, J. Pate, R. Hooker, SP. The contribution of dance to daily physical activity among adolescent girls. *Int J Behav Nutr Phys Act*, 2011; 8:87.

36. Nader PR, Bradley RH, Houts RM, McRitchie SL, O'Brien M: Moderate-to-vigorous physical activity from ages 9 to 15 years. *JAMA* 2008, 300:295-305.

37. Alvarez-Farizo B, Hanley N, Barberan R. The value of leisure time: a contingent rating approach, *J Environ Plan Man*, 2001; 44,5:681-699.

For peer review only

38. Ridyard C, Hughes D. Methods for the collection of resource use data within clinical trials: a systematic review of the studies funded by the UK Health Technology Assessment Programme. *Value Health*, 2010; 13, 8:867-872.

TABLES

Table I. Attributes and levels of the Discrete Choice Experiment and the four choice sets given to participants

Level of attributes	Attributes		
	Frequency of dance classes per week	Cost per session	Hours left for other leisure activities on that day
**Upper**	2 dance classes per week	£1 per session	Leaving 3 hours for other leisure activities on the evening of the dance session
**Lower**	1 dance class per week	50p per session	Leaving 2 hours for other leisure activities on the evening of the dance session

1A<br>You take one after school dance class each week at a cost of £1 per class leaving you 3 hours on that evening for other leisure activities	1B<br>You take two after school dance classes each week at a cost of 50p per class leaving you 2 hours on those evenings for other leisure activities
2A<br>You take two after school dance classes each week at a cost of £1 per class leaving you 2 hours on those evenings for other leisure activities	2B<br>You take one after school dance class each week at a cost of 50p per class leaving you 3 hours on that evening for other leisure activities
3A<br>You take one after school dance class each week at a cost of 50p per class leaving you 2 hours on that evening for other leisure activities	3B<br>You take two after school dance classes each week at a cost of £1 per class leaving you 3 hours on those evenings for other leisure activities
4A<br>You take two after school dance classes each week at a cost of 50p per class leaving you 3 hours on those evenings for other leisure activities	4B<br>You take one after school dance class each week at a cost of £1 per class leaving you 2 hours on that evening for other leisure activities

For peer review only

For peer review only

Table II. Resources use identification template used to inform BGDP feasibility study

NRG Youth Dance & Health Project[1]	
	[2]*Total Cost £*
Project planning work: initial research into existing action research projects	500.00
Lead artist fee -programme design/artist training	800.00
Artists' travel fees – attending training/planning sessions	637.35
Artists' fee	5,806.00
Artists' travel costs	1,515.44
Coach hire – school group for pilot session	562.88
Space hire	254.70
Disclosures/refreshments	77.98
Postage	64.03
Management fee	4,000.00
Staff travel	443.65
Documentation (dissemination advocacy)	269.70
Road-show event – end of project	562.88
Additional schools workshop	151.80
Total 2005-6 prices £	15,203.53
Teacher time for behaviour management (not included in NRG report)[3]	3,300.00
Total 2007-8 prices £ with teacher management	19,427.76
Total 2010-11 prices £	20,600.00

[1]resource use items identified by Hampshire Dance and Laban, NRG Youth Dance and Health Project Evaluation report[20]
[2]assumptions and costing profile produced by Fordham & Barton (2008)[18] for NICE Guidance 17 (NICE,2009)[19]
[3]This item was identified in the NICE report [20] but not costed [18 19]

For peer review only

For peer review only

Table III. Description of resources used, unit volumes, prices and estimated costs* by category by school by pupil in 2010-11 prices

Stage of BGDP	*Description of resources used*	*Number of units*[1]	*Price per unit*[2]	*Cost £ 2010-11 prices*	*Cost stage*
Pre-Programme planning development stage **0**	Lead dance artist consultation and development work	2 days	£500 per day	1000	
	Lead dance artist time, to adapt training programme for hip hop genre	1 day	£500 per day	500	
	Lead dance artist time, to prepare dance teachers for 9 week intervention	1 day	£500 per day	500	
	3 dance teachers preparation/training time	7.5 days	£27 per hour	2400	
	Travel expenses			288[3]	
	Disclosures CRB forms			38[3]	4726
Programme preparation stage **1**	Space hire for dance taster sessions in intervention schools	6 hours	£15 per hour	90	
	Dance teacher delivery of taster sessions in intervention schools	6 hours	£27 per hour	162	
	Control schools (n=4) recruitment presentations	1 day	500 per day	500	
	Postage costs			25[3]	
	Travel expenses			42[3]	819
Programme delivery stage **2**	Delivery 18 dance classes at 90 minutes per class over 9 weeks x 3 schools	81 hours	£27 per hour	2187	
	T-shirts for 3 intervention schools	90 girls	£5 per T-shirt	450	
	Small gift incentives for control schools data collection	3 waves	£731 per wave	2192	
	Refreshments			100[3]	
	Travel expenses			446[3]	5375
Programme reinforcement stage **3**	Half-day dance workshops for two control schools	9 hours	£27 per hour	243	
	6 hour performance events for parents of girls at 3 intervention schools	18 hours	£27 per hour	486	729
				Total	£11,649

BGDP Stages 0-3 costs £s	£11,649 2010-11 prices
BGDP Stages 1-3 costs £s	£6,923 2010-11 prices
BGDP Stages 1-3 minus control costs £s	£3,988 2010-11 prices
BGDP cost per school £s	£1,329 2010-11 prices
BGDP cost per pupil £s	£44.31 2010-11 prices

*Research team administration, travel and other costs are not included. Control costs in this research are included for information
Sources: 1. research team 2. project budget - all prices are actual rates paid. 3. Project budget - all costs are actual expenses incurred.

For peer review only

Table IV. Preference rankings of first choice leisure activities at each time point N (%)

	Time 2		Time 1		Baseline Time 0	
After-school leisure activity	*Ranking*	*N (%)*	*Ranking*	*N (%)*	*Ranking*	*N (%)*
Go around with friends to shopping centres, streets, parks just for fun	1	46 (26)	1	33 (25)	2	12 (18)
Use the internet for fun: chats, YouTube, Facebook, Bebo, Myspace, looking for music (do not include school homework)	2	31 (17)	3	20 (15)	3	8 (12)
Take part in sports, athletics or physical activity	2	31 (17)	2	22 (17)	1	13 (20)
Play with or see friends at your home or their homes	3	21 (12)	4	11 (9)	5	5 (7)
Read books for enjoyment (do not include school books)	4	13 (7)	5	10 (8)	4	6 (9)
Go to discos or dance classes	5	11 (6)	8	5 (4)	5	5 (7)
Play a musical instrument, sing, draw, paint or write	6	9 (5)	4	11 (9)	3	8 (12)
Send text messages or use Twitter on your mobile phone	7	8 (5)	7	6 (5)	5	5 (7)
Play computer games	8	4 (2)	6	8 (6)	6	4 (6)
Watch TV DVDs or playbacks of programmes	8	4 (2)	9	4 (3)	7	2 (3)
Total of valid* responses/total responses		178/210		130/210		68/210

*A valid response = each after-school leisure activity is ranked by a separate number between 1 and 10 by each individual participant using a PDA

For peer review only

Table V. Regression coefficients indicating the value of dance classes at t0 (week 0) and t1 (week 9) by group allocation

	Control time 0 (n=104/120)*			*Control time 1 (n=104/120*)*			*Intervention time 0 (n=80/90*)*			*Intervention time 1 (n=80/90*)*		
	Coeff	SE	*P value*	Coeff	SE	*P value*	Coeff	SE	*P value*	Coeff	SE	*P value*
**Frequency of dance class:**			*<0.01*			*<0.01*			*<0.01*			*0.04*
Twice a week	**0.18**	0.07		**0.30**	0.07		**0.25**	0.58		**0.13**	0.06	
Three times a week	-0.18	0.07		-0.30	0.07		-0.25	0.58		-0.13	0.06	
**Cost:**			*<0.01*			*<0.01*			*<0.01*			*<0.01*
£1	**0.22**	0.05		-0.17	0.04		**0.46**	0.07		**0.26**	0.06	
£2	-0.22	0.05		**0.17**	0.04		-0.46	0.07		-0.26	0.06	
**Other hours available for leisure activities on dance class days:**			*<0.01*			*<0.01*			*<0.01*			*<0.01*
2 hours	**0.35**	0.06		-0.31	0.65		**0.76**	0.11		**0.37**	0.08	
3 hours	-0.35	0.06		**0.31**	0.65		-0.76	0.11		-0.37	0.08	

*Preferred level of attribute in bold, attribute most concerned with shaded dark grey, attribute least concerned with shaded light grey *Number of valid responses from total possible responses*

For peer review only

For peer review only - http://bmjopen.bmj.com/site/about/guidelines.xhtml

RESEARCH PILOT STUDY

Bristol Girls Dance Project feasibility study: using a pilot economic evaluation to inform design of a full trial

Powell, J.[1§] Carroll, FE.[2] Sebire SJ.[3] Haase, AM.[3] Jago, R.[3]

[1] Professor of Public Health Economics, Department of Health and ~~Applied~~ Social Sciences, University of the West of England, Glenside Campus, Stapleton, Bristol, BS16 1DD, UK.

[2]Dr Fran E Carroll, Research Assistant, School of Social and Community Medicine, University of Bristol, BS8 2PS, UK.

[3]Dr Simon J Sebire, Post-Doctoral Researcher, Centre for Exercise Nutrition and Health Sciences, School for Policy Studies, University of Bristol, BS8 1TZ, UK.

[3]Dr Anne M Haase, Senior Lecturer in Exercise, Nutrition & Health, Centre for Exercise, Nutrition and Health Sciences, School for Policy Studies, University of Bristol, BS8 1TZ, UK.

[3]Professor Russell Jago, Professor of Paediatric Physical Activity & Public Health Exercise, Nutrition & Health Sciences, Centre for Exercise, Nutrition & Health Sciences, School for Policy Studies, University of Bristol, BS8 1TZ, UK.

[§]Corresponding author Professor Jane Powell, Department of Health and ~~Applied~~ Social Sciences, University of the West of England, Bristol, BS16 1DD, UK. 0044 117 3288752

For peer review only

ABSTRACT

Wider context: There is currently little guidance for pilot trial economic evaluation where health outcomes and costs are influenced by a range of wider determinants and factors.

Objectives: This paper presents the findings of a pilot economic evaluation study running alongside the Bristol Girls Dance Project (BGDP) feasibility study~~ and describes how these have impacted the conduct and design of main trial economic evaluation~~.

Design: Three-arm, cluster randomised, controlled pilot trial and economic evaluation. Seven schools (n=210) from the Bristol and greater Bristol area, UK were randomly allocated to the intervention arm 3 schools (n= 90) and the control arms 4 schools (n=120).

Intervention: Girls aged 11-12 years with parental consent were provided with two, 90-minute dance sessions per week for 9-weeks at school facilities.

Economic outcome measures: Programme costs~~,~~ and girls' preferences for attributes of dance and preferences for competing leisure time activities were measured. ~~Primary outcome: accelerometer-derived moderate-to-vigorous physical activity (MVPA) minutes per day.~~

Results: ~~After-school dance classes for girls aged 11-12 years are valued favourably in terms of other competing leisure choices compared with older adolescents. Discrete Choice Experiment (DCE) methods are acceptable to girls of this age.~~ The mainstream average cost of the BDGP programme (not including research, control and dance teacher training costs) per school ~~not including developmental and research costs at mainstream implementation~~ ~~is~~was $~~2,555~~2,126.40; £~~1,596~~1,329; €~~1,870~~1,555 and per participant $~~85.17~~70.90; £~~53.21~~44.31; €~~62.32~~51.84 in 2010-11 prices. Discrete Choice Experiment (DCE) methods are acceptable to girls of this age indicating time available for other leisure activities on

For peer review only

dance class days is the attribute girls valued most and 2 hours leisure time remaining preferred to 3 hours.

.

Conclusions: This pilot study indicates The feasibility of pproviding full cost data for a fullture trial of the BGDP programme is feasible and practical. There is no evidence from preference data to support adjustment to intervention design. A future economic evaluation is likely to be successful utilising the resource use checklist developed. has been established and an embryonic resource use checklist developed for this purpose. The importance of categorising separately This pilot study has demonstrated resources used to develop, prepare and, deliver and maintain the programme should be categorised separately, so the cost of the mainstream programme can be to estimated mainstream costs accurately is demonstrated in a full trial.

Strengths and limitations of this study

This pilot study used a systematic approach where there is currently minimal evidence to determine consider how the conceptual and measurement process of an economic evaluationthe costs of implementing a pilot dance intervention in girls 11-12 years old. might be enhanced in a full trial by learning more about the cost and outcome dimensions of economic evaluation and has The study has produced findings about girls' preferences for dance and an embryonic costing tool that can be applied to design and conduct an economic evaluation alongside a full cluster RCT.

This feasibility and exploratory pilot study is powered to test the intervention concept, the feasibility of obtaining programme cost data in categories and the evidence required to power

For peer review only

a full cluster RCT in the future. Consequently, the variation in programme costs at the school level has not been captured and this is a limitation of the ~~programme~~ cost estimates presented.

~~A social model of costing that reflects opportunity costs of participants and parents could be considered.~~

BACKGROUND

Recent influential studies attach substantial economic and social costs to obesity prevalence projections.[1 2] These forecasts are based upon a body of research from long term cohort studies which suggest that change in the prevalence of obesity in children and adolescents born at the millennium is likely to lead to increased health risks in middle-age irrespective of adult adiposity.[3 4 5] Consequently, there is a need for new interventions that focus on preventing obesity or changing diet or physical activity; the two behaviours that are central to the accrual of body mass.

As well as being a health and wellbeing issue, children's obesity also has serious economic impacts. Scarce resources with competing uses in all health systems and the need to decide between new, 'efficacious' primary prevention physical activity programme interventions on the grounds of cost-effectiveness, has increased the significance of economic evaluation as a concept and methodology. Recent guidance from the UK Medical Research Council (MRC) for the development and evaluation of complex behavioural interventions suggests that efficacy and cost-effectiveness should be established before programmes are implemented at the population level.[6 7] However, the meaningful determination of these criteria is often problematic in primary prevention and guidelines for the design and conduct of economic evaluation of complex interventions are at an early stage of development.[8 9 10 11] It is therefore

For peer review only

important to develop the conceptual and measurement process by which effectiveness and cost-effectiveness of complex physical activity interventions can be evaluated in a full trial using a pilot study.

The main findings of the Bristol Girls Dance Project (BGDP) feasibility trial~~,~~ concerning process evaluation, outcomes and effectiveness have been published elsewhere ~~examined the potential utility of an after-school dance programme as a means of increasing moderate to vigorous physical activity (MVPA) among 11-12 year old girls (school year 7) seeking to discover any improvements that might need to be made to the programme prior to a main trial~~.[12] This part of the study suggested that it is feasible to deliver the intervention and that participating in dance has the potential to yield change in moderate to vigorous physical activity (MVPA) among 11-12 year old girls (school year 7) ~~girls' physical activity~~, but a larger randomised controlled trial (RCT) would be needed to fully evaluate its effectiveness and cost-effectiveness.[12] In the absence of ~~other~~robust evidence for the cost and outcome of dance interventions, ~~an~~other aims of the feasibility pilot w~~as~~ere to refine the information required to sufficiently power a full trial and to use the preference data to inform potential refinements to intervention design.

Preferences for competing after-school activities are potential determinants of the economic benefit of dance intervention, as increased physical activity must be valued in order for it to be maintained [8] and to have potential for long-term impact on physical activity levels. In this study, Discrete Choice Experiment (DCE) and survey methods are applied to examine two separate, but complementary aspects of value –preferences for the attributes of dance classes and preferences for dance among other competing alternatives for spending leisure time respectively. Physical activity levels decline during youth [13] with the start of secondary school being a critical period of change, so it was important to establish comparative preferences for after-school, leisure activities on weekdays.

For peer review only

Value is a concept germane to recruitment and retention rates and linked to the outcome dimension of the BGDP intervention and therefore important to examine in detail. Discrete Choice Experiment (DCE) works on the premise that any 'product', for example a healthcare treatment or physical activity programme, can be described by its characteristics, or attributes, and the extent to which an individual values a 'product' is dependent on the level of these characteristics. [14 15 16]

Thus, this paper reports the learning from and findings of a pilot economic evaluation of the Bristol Girls Dance Project for girls aged 11-12 years in a primary school setting in England impact the that can be applied to design and conduct of a future full trial and economic evaluation. Other findings from the Bristol Girls Dance Project Feasibility Trial concerning process evaluation, outcomes and effectiveness have been reported elsewhere.[12]

ARTICLE SUMMARY

Article focus

To examine whether the feasibility of proposed methods for collection of resource use data for estimating the costs are feasible, practical and likely to be successful in full trial economic evaluationproviding complete programme cost data from a funder perspective.

To understand preferences for after-school dance classes in the context of weekday leisure time at age 11-12 years that can be applied to inform intervention design. To demonstrate the appropriateness of using DCE and survey methods to derive participants' preferences for dance among other competing leisure activities.

To present learning from reflections on the findings of pilot economic evaluation to inform a full trial economic evaluation

For peer review only

Key messages

Time available for other leisure activities on dance class days is an attribute of after school dance classes girls aged 10-11 years valued most - followed by cost and frequency of dance classes per week.

2 hours available for other leisure activities on dance class days was preferred to 3 hours suggesting after-school dance classes are valued compared with other ways to spend leisure time after school on weekdays

Resources used in the development, preparation, delivery and maintenance of dance classes should be categorised separately in stages in order to identify the mainstream cost of the programme intervention to commissioners

A pilot economic evaluation has been applied to learn more about the feasibility of costing the BGDP complex intervention and categorising programme resources to identify the mainstream cost of the intervention during a full trial.

Pilot data from seven schools has been analysed to understand girls' preferences for dance versus other ways of spending their leisure time at age 11-12 years that can help to conceptualise and measure the outcome dimensions of economic evaluation alongside a full trial.

METHODS

Bristol Girls Dance Project (BGDP) feasibility study

For peer review only

BGDP was a three-arm, parallel group, cluster randomised, controlled pilot trial with schools as the unit of allocation. Seven schools from three school districts, Bristol, Bath and South Gloucestershire (UK) were recruited to take part in the study from schools in these districts with no current after school dance provision.[12] The hip-hop and street dance style of dance to popular music was facilitated by a professional dance teacher.

Stratifying by school district, three schools were randomly allocated to the intervention arm (n= 90) and four schools to the two control arms (n=120) and each school was assigned a dance teacher to lead the sessions. Randomisation was conducted by an independent member of the clinical trials unit at Bristol University using computer-generated random sequences and codes for school district and school name. A full description of thiha been detailed in another paper from this study. [12] The three intervention schools received two, 90-minute after-school dance classes per week for 9 weeks selected to allow the entire programme to be delivered within a school term. Pilot work had suggested that dance is a very attractive form of PA for girls, so the control element was designed to ascertain whether offering a dance workshop at the end of the research process (i.e., after the last data collection), would affect either retention or the quality of data provided by participants. We therefore utilized a three-arm design with two different control groups. In two schools, participants were provided with small thank you gifts for each wave of data collection. In the other two control schools participants were provided with the same small thank you gifts, as well as a half-day dance workshop at the end of the study.

Sample size

For peer review only

This feasibility study was powered to test the intervention concept and to provide the necessary information to calculate the sample size of a full cluster RCT and economic evaluation of an after school dance programme. The important parameters detection of 10 minutes difference in MVPA per weekday (50 minutes per week) between the intervention and control groups, intra-class correlation for weekday MVPA at time 2 and associated confidence intervals have been reported and profiled in another paper from this study. [12] For practical reasons the sample was limited to thirty girls aged 11-12 years per school. Girls were recruited from each school at random from those with parental consent.

The intervention

Intervention participants were provided with two, 90-minute dance sessions per week for 9 weeks at school facilities. The hip-hop and street dance style of dance to popular music was facilitated by a professional dance teacher.

Primary outcome measure

All participants were asked to wear an Actigraph accelerometer for seven days at baseline (week 0) during the last two weeks of the intervention (weeks 8 or 9) and 3 months after the intervention ended (Week 20).

For peer review only

Economic measures

1) Resource use-cost

Resources used at each stage of the BGDP programme were recorded retrospectively by the project team on time-sheets and expense sheets from a public sector perspective. Costing followed a method applied in a study of complex intervention with children in a school setting (A Stop Smoking in Schools Trial) ASSIST.[17] Stage 0 intervention planning and development costs or costs associated with running the scientific study (e.g., control group incentives for data collection, control school dance workshops or recruitment events) would not recur during mainstream implementation, but these costs are included for completeness.

Expenses including entry incentives, postage and Criminal Records Bureau (CRB) checking were gathered. Travel time was estimated for all staff inputs at the school locations. Girls in the control schools received incremental thank you gifts at each data collection they attended. Space hire did not incur costs, but estimates of the cost of space hire for dance class delivery are included because costs connected with alternative use of space in schools. School overhead and capital costs are not included.

2)1) Discrete Choice Experiment (DCE) and survey of preference ranking and use of leisure time

BGDP formative qualitative work indicated the frequency of after-school dance classes per week;, cost per session and how much leisure time is left over on dance class days for other leisure activities are important considerations for girls in deciding whether to participate.[187] Participants were asked to select the 'dance class scenario' they preferred from a pair of

For peer review only

options. Table I presents the 4 paired scenarios (1A:1B, 2A:2B, 3A:3B, 4A:4B) consisting of a randomly determined combination of three attributes, each with two levels.

Table I

Four paired choice scenarios were administered to 210 girls in seven schools - 3 intervention schools (n=90) and 4 control schools (n=120). Measures were taken at baseline (time 0) and at 9 weeks (time 1) using large cards, and girls' preferred choice for each pair of scenarios was recorded by the project team. Two time points were needed to establish change in preferences before and after the intervention. Participants were also asked to give preference ratings for ten leisure activities on weekdays by survey using a ten-point scale (1 = favourite; 10 = least favourite). Participant responses were collected on Personal Digital Assistants (PDAs) and downloaded to a customised database.

2) Resource use-cost

At commencement of this pilot study there was minimal evidence on which to draw in identifying costs that might be included in a resource use checklist. One report from the National Institute for Health and Clinical Excellence (NICE) had modelled the cost of delivering dance classes to young children and produced some ball park cost estimates.[18 19] These were based on an account of the resources used in delivery of a dance programme for girls by Hampshire Dance and Laban[20] in which resources had been identified, but not costed. These uncontrolled studies provided a starting point and an opportunity to produce more complete and accurate costing data from the Bristol Girls Dance Project feasibility pilot trial in which the volume of resources used and prices could be treated separately. The cost items identified by NICE were entered into a database and data collected using time-sheets and expense sheets were collected by the project team. These cost estimates and some

For peer review only

estimates for teacher time to manage behaviour derived by the first author of this paper are detailed in Table II.[18 19 20] Table II was used as a template for identifying and costing resources in the BGDP feasibility study.

TABLE II here

Ethics

Potential participants in all seven schools were told that there was a maximum of 30 randomly assigned spaces at the dance classes. The study was approved by a University of Bristol ethics committee and informed parental consent was obtained for all participants.

Analyses

Proportions of the sample ranking ten weekday leisure activities as first choice activity (rank = 1) were calculated after participants had rated all ten leisure activities from 1 to 10. Responses from participants with repetition of ratings for one or more leisure activities or missing ratings for leisure activities were excluded. Overall, where the proportion of the sample rating activities as their first choice was the same, these activities were assigned the same rank across all ten activities. ~~Valid responses as a proportion of total responses for the survey ranking leisure activities were t2=178/210, t1=130/210, t0=68/210 across all group allocations.~~

DCE data was 'effects-coded' [~~19~~21] using STATA [2~~0~~2] and analysed using conditional logistic regression. Effects coding is similar to dummy variable coding, but is preferable in this instance because interaction or trade-off between the attributes is likely to take place as well

For peer review only

as a main effect. The coefficients for each attribute are a measure of the influence of that attribute level on choice. Positive values represent a positive influence on choice, or in other words, a preference for that level of an attribute. These results can be used to establish girls' overall preferences for attributes, as well as the order of their preferences (i.e. which attribute is most and least important). Participants with missing data were excluded from the DCE analysis.

Total and average cost estimates from a funder perspective were identified and derived for BGDP based on staged timing, quantity, frequency and price of resource use in 2010-11 prices. Expenses including travel, intervention programme entry incentives, postage, Criminal Records Bureau (CRB) applications were accessed from the database kept by the project team. Girls in the control schools received small thank you gifts at each data collection they attended. Space hire did not incur costs, but estimates of the cost of space hire for dance class delivery are included because they are costs connected with alternative use of space in schools. School overhead and capital costs are not included.

Grouping costs to enable estimation of the mainstream cost adopted the categories used in (A Stop Smoking in Schools Trial) ASSIST.[23] Stage 0 intervention planning, development and training costs, stage 1 intervention preparation, stage 2 intervention delivery and stage 3 intervention maintenance costs were separately identified. Training costs for dance teachers are identified separately. Costs associated with running the research study, control group incentives for data collection, control school dance workshops and recruitment events) would not recur during mainstream implementation, but these costs are included for clarity and completeness. All costs connected with tasks undertaken by the research team are not included.

For peer review only

RESULTS

Identification and timing of resources used

Table III identifies and describes at four stages the resources use of the BGDP programme and presents total cost estimates. The proportion of total costs incurred were 4~~2~~1% at stage 0, ~~5~~7% at stage 1, 4~~7~~6% at stage 2 and 6% at stage 3. ~~BGDP required at least 286 hours of staff input.~~ At stage 0 half of the costs are dance teacher preparation and training time which arguably would be incurred in part in delivery of a mainstream form of the programme. Eighteen BGDP dance classes (2 classes per week for 9 weeks) of 90 minutes duration were delivered to 90 girls in three intervention schools (30 per school) for 81 hours (27 hours per school) at a total estimated cost ~~of $7,666; £4,789; €5,609~~$6,380, -£3, 988, €4,666 in 2010-11 prices.[24~~1~~] [25~~2~~] ~~Removing stage 0 programme development and stage 1-3 research costs revealed an~~ The average cost of ~~repeating a~~the BDGP programme in its mainstream form per school ~~of~~ was $2,126.40; £1,329; €1,555 and per participant $70.90; £44.31; €51.84 ~~$2,555; £1,596; €1,870 and per participant of $85.17; £53.21; €62.32~~ in 2010-11 prices. If training costs for dance teachers on the BGDP were included to the mainstream cost this would add $1280; £800; €928 to the cost per school and $43; £27; €31.60 to the cost per pupil. These are not insubstantial additions, but are at the high end of training costs because this new dance programme was properly prepared for delivery. Training costs for the delivery of an established dance programme are likely to be lower. It was not possible to calculate ~~There are no~~ confidence intervals for ~~these~~ average programme costs- per school or per participant~~or participant estimates~~ (see limitations of this pilot study).

Table III here

For peer review only

The BGDP programme compares favourably in terms of average cost per user with the only other evidence for costing an after-school dance class programme in England - estimated by Fordham and Barton [23] and included in NICE guidance [24] on the promotion of physical activity in children. These estimates [23 24] were based on a report of the NRG dance programme [25] which was delivered at a total cost of $32,975; £20,600; €24,129 at 2010-11 prices. Assumptions were made [23] to derive costs for NICE guidance from the NRG report.[25] The shorter and less intensive NRG programme costed in the NICE report [19] comprised 10 dance classes of 60 minutes duration for an assumed number of 24 girls (an assumed number) in 14 groups or schools (n=336 girls) for 140 hours with a cost per participant of $98.14; £61.31; €71.81, in 2010-11 prices. This includes 140 hours of teacher time sourced from national pay scales for England at £23.57 per hour at 2010-11 prices.[26]

Preferences for leisure activities

All girls were asked to rank ten after-school leisure activities by first preference activity. Valid responses as a proportion of total responses for the survey ranking leisure activities were t2=178/210, t1=130/210, t0=68/210 across all group allocations.

Table III presents proportions across the participants (n=210) for preference ranks for after-school leisure activities for all group allocations at each time point demonstrating consistency in preference ranks indicating girls' selection of first choice leisure activities at each time point. The after-school leisure activities indicating the highest proportion of first choice preference rankings at each time point include 'hanging out with friends away from home just for fun' (Ranking at t2=1, t1=1, t0=2); 'take part in sports, athletics or physical activity' (Ranking at t2=2, t1=2, t0=1) and 'using the internet for fun: chats, YouTube, Facebook, Bebo, Myspace, looking for music' (Ranking at t0=3, t1=3, t2=2). -Valid responses were

For peer review only - http://bmjopen.bmj.com/site/about/guidelines.xhtml

included in the analyses. Valid responses as a proportion of total responses for the survey ranking leisure activities were t2=178/210, t1=130/210, t0=68/210 across all group allocations indicating particularly at baseline the participants experienced some problems using a hand held PDA to rank and rate the weekday after-school leisure activities.

Table IIIV here

DCE results

The p values for the regression coefficients in Table IV indicates girls in this sample have a preference for "time left for other leisure activities on dance class days", over the "cost of" and "frequency of dance classes per week". Analysis of preference levels within each attribute suggests 2 hours is preferred to 3 hours remaining for other leisure activities on dance class days. This pattern was consistent in all intervention and control groups at t0 and t1. Girls were least concerned with the frequency of dance classes per week with preference proportions suggesting 2 classes were preferred to 1 dance class per week in both intervention groups and the baseline control group. This pattern was consistent in all groups at t0 and t1.

Table IV here

DISCUSSION

What is already known on this topic

There is minimal guidance to support how economic evaluations of complex public health interventions should be designed and conducted in school and community settings.

For peer review only

There are no checklists or tools available to support costing dance programmes and minimal knowledge of how to categorise resources to identify the mainstream cost of delivery.

Discrete Choice Experiment (DCE) methods to elicit the relative preferences and choices of girls aged 11-12 years are untried and untested, but it is important to capture how girls value dance among other competing leisure activities using a robust and acceptable method.

What this study adds

Providing programme cost data for a full trial of the BGDP programme is feasible, practical and likely to be successful. Around two-fifthsthirds of resources are development and research control costs, so resources used to develop, prepare and deliver these programmes should be categorised separately, so the cost of the mainstream programme can be estimated accurately in a full trial.

Discrete Choice Experiment is an acceptable method to elicit preferences of girls aged 11-12 years.

At this point in their lives after-school dance is an activity valued by popular choice girls when offered within the context of other competing choices and parental support for activities for spending leisure time after school on weekdays. predicts positive recruitment and retention rates in after-school dance activities.

Participation in after-school dance classes has opportunity costs for participants and parents extending beyond the funder that suggest a social model of cost should be considered for a full trialto capture the costs associated with intervention outcome.

For peer review only

Robust evidence for the cost-effectiveness of physical activity complex interventions is important for knowing where to invest scarce resources and commission programmes to maximise health outcomes in primary prevention.[27 28 29] However, gathering robust evidence to support investment in public health interventions is a challenge.[30 31] Significant barriers remain and there is currently little guidance in how to conduct economic evaluation where behaviour change is associated with health outcomes determined beyond genetic inheritance by family, social and physical environments. [32 33]

Indicative programme cost data from the pilot economic evaluation indicated a substantial proportion of the intervention programme costs 4~~2~~1% occur at Stage 0 – the pre-programme ~~planning and~~ development stage. This is an important finding because it suggests provided BGDP is effective and cost-effective in a full trial, it would be substantially less costly to roll out in its mainstream form. All complex interventions in primary prevention are likely to generate a high proportion of upfront development costs that will not reoccur once a programme is mainstreamed – an aspect of investment in public health interventions often overlooked by decision makers.

Application of DCE is an established technique in adult populations, but to our knowledge has not been applied previously in populations of children aged 11-12 years to establish values for the attributes of physical activities. This study has demonstrated application of DCE methods is feasible and acceptable to girls of this age. This is important because it suggests DCE could be applied in other studies with children to understand the concept of 'value' of an activity which plays an important role in recruitment, participation and maintenance of participants which are all linked to intervention outcome. In addition to its acceptability in this study the DCE method has produced more complete and valid data than the direct survey method in eliciting preference ranks for after-school leisure activities. These

For peer review only

findings support a previous contention that DCE techniques may have merit over more 'traditional' survey methods [34] in eliciting preferences. However, more evidence would be required to fully support this finding.

Taken together, findings of the DCE and survey of leisure activity preference in this study indicate dance is a valued leisure activity among competing alternatives and reveals more about the attributes of dance classes in girls of this age that can be taken forward to maximise recruitment and retention in the BGDP programme. The findings of this study suggest that dance has immediate appeal as an after-school leisure activity among a range of strongly competing alternatives in girls of this age compared with older adolescents.[35] Girls in this study have a first rank preference for the attribute 'time remaining for other leisure activities on dance class days', over the 'cost of' and 'frequency of dance classes per week'. The finding that in the intervention group two hours is preferred to three hours remaining for other leisure activities on dance class days is significant. Overall these findings could suggest that at this point in their lives dance is ~~popular choice in~~ valued by girls as a physical and social activity ~~of this age~~ when offered within the context of ~~other~~ competing and constrained choices for spending leisure time at this age. For example, at this age girls are not likely to be able to go to 'discos or dance classes' without parents or carers or to 'hang around on street corners with friends' and these issues may have affected their responses in the survey. These are important findings because they predict positive recruitment rates and participation of girls aged 11-12 years in dance as a physical leisure time activity and in a full trial. [36]

Delivery of after-school dance classes is dependent upon substantial commitment from the girls giving up their after-school leisure time to participate in dancing. In turn, participation is dependent upon the willingness of parents and carers to support attendance and to provide encouragement and a means of travelling back home after school hours when school buses are not available. This pilot study suggests development of a social model of costing that

For peer review only

reflects the cost of participants' and parents' time and opportunity costs are substantial elements of the intervention cost that could be captured, if practical, in a full trial.

However, methods and tools to capture 'hidden' cost items that facilitate the success of the intervention, but are not incurred by funders are not yet fully established. [~~17~~23] Where to include training costs in these metrics is a question that remains for a future trial as they should arguably be included in mainstream cost estimation despite their categorisation as development costs. How identification of costs falling outside the public sector that are relevant to programme implementation can be captured at a full trial stage also needs to be considered carefully.[37] In a full trial resources used should be captured prospectively [38] and this pilot study has established categories of resource use are also important to consider to establish accurate mainstream programme costs.

CONCLUSIONS

The feasibility of providing costing data for full trial of the BGDP programme in is established and an embryonic resource use checklist has been developed. Resources used to develop and run the BGDP programme should be categorised separately in order for the mainstream delivery cost of BGDP to be estimated accurately in a full trial. A social model of costing that reflects participants and parents opportunity costs should be considered. BGDP after-school dance classes have potential for sustained participation and cost-effective delivery, but a full trial using methodological learning from this study is required.

Competing interests

There are no competing interests.

For peer review only

Authors' Contributions

The quantitative analysis plan was conceived by JP, FC, RJ and SJS. Analyses were performed by JP and FC and interpretation by JP, FC, RJ, SJS and AMH. JP drafted the first version of the manuscript with additional sections provided by FC. All authors provided critical edits and revisions to the paper and have reviewed and approved the final version of the paper.

Data sharing statement No additional data are available

Funding

This research was funded by the National Prevention Research Initiative (http://npri.org.uk), consisting of the following funding partners: Alzheimer's Research Trust; Alzheimer's Society; Biotechnology and Biological Sciences Research Council, British Heart Foundation; Cancer Research UK; Chief Scientist Office; Scottish Government Health Directorate; Department of Health; Diabetes UK; Economic and Social Research Council; Engineering and Physical Sciences Research Council; Health & Social Care Research & Development Office for Northern Ireland; Medical Research Council; The Stroke Association; Welsh Assembly Government and World Cancer Research Fund [grant number G090084].

Acknowledgments

We would like to thank all the participants, dance teachers, school teachers and schools who participated in or assisted with this research. Professor Powell acknowledges discussion of and feedback from a draft version of this paper presented at the Health Economists Bristol (HEB) journal club http://www.bris.ac.uk/social-community-medicine/centres/healthecon in

For peer review only

November 2011. Laura Davis project manager BGDP and Jade McNeil research associate are acknowledged for data collection leading to this paper. Fran Carroll's contemporaneous PhD research was supervised by Dr Alan A Montgomery at the time of DCE design and data analysis. Dr Jago is supported by a National Institute for Health Research career development fellowship. This work was also supported by DECIPHer, a UKCRC Public Health Research: Centre of Excellence. Funding from the specific funders under the auspices of the UK Clinical Research Collaboration is gratefully acknowledged. The other co-applicants of the main BGDP study, Professor Ashley R Cooper and Dr Alan A Montgomery are acknowledged. The views expressed in this publication are those of the authors and not necessarily those of the NHS, the National Institute for Health Research or the Department of Health.

For peer review only

For peer review only

References

1. Foresight. *Tackling obesities: future choices.* London: Government Office for Science, 2007.

2. Stamatakis E, Zaninotto P, Falaschetti E, Mindell, J. Head J. Time trends in childhood and adolescent obesity in England from 1995 to 2007 and projections of prevalence to 2015. *J Epidemiol Community Health* 2010; **64**:167-174.

3. Gunnell D, Frankel S, Nanchahal K, et al Childhood obesity and adult cardiovascular mortality: a 57 year follow-up study based on the Boyd Orr cohort. *Am J Clin Nutr* 1998: 67:1111-18.

4. Must A, Jacques PF, Dallal GE, et al. Long term morbidity and mortality of overweight adolescents – a follow-up of the Havard growth study of 1922 to 1935. *N Engl J Med* 1992: 327:1350-5.

For peer review only

5. Engeland A, Bjorge T, Sogaard AJ, et al. Body mass index in adolescence in relation to total mortality: 32-year Follow-up of 227,000 Norweigan boys and girls. *Am J Epidemiol* 2003:157:517-23.

6. Lancaster GA, Dodd S, Williamson P. Design and analysis of pilot studies: recommendations for good practice. *J Eval Clin Pract* 2004, 10, 2:307-312.

7. Craig P, Dieppe P, Macintyre S, Michie S, Nazareth I, Petticrew M. Developing and evaluating complex interventions: the new Medical Research Council guidance. *BMJ* 2008, 337:a1655.

8. Wolfenstetter SB. Conceptual framework for standard economic evaluation of physical activity programmes in primary prevention. *Prev Sci.*2011; 12(4):435-51.

9. Sheill, A. Hawe, P. Gold, L. Complex interventions or complex systems? Implications for health economic evaluation. *BMJ*, 2008; 336:1281-1283.

10. Weatherly H, Drummond M, Claxton K, et al. *Assessing the challenges of applying standard methods of economic evaluation to public health interventions.* York: Centre for Health Economics, 2009.

11. Anderson R. Systematic reviews of economic evaluations: utility or futility. *Health Econ*, 2010;19:350-364.

12. Jago R. Sebire S, Cooper AR, et al. Bristol Girls Dance Project Feasibility Trial: Outcome and process evaluation results. *Int J Behav Nutr Phys Act*, 2012; 9, 83.

For peer review only

13. Gorely T, Marshall S, Biddle SJH, Cameron N. The prevalence of leisure time sedentary behaviour and physical activity in adolescent girls: An ecological momentary assessment approach. *Int J Pediatr Obes*, 2007; 2:227-234.

14. Cheraghi-Sohi S, Bower P, Mead N, McDonald R, Whalley D, Roland M. Making sense of patient priorities: applying discrete choice methods in primary care using 'think aloud' technique. *Fam Pract*, 2007; 24: 276-282.

15. Green C, Gerard K. Exploring the social value of health-care interventions: a stated preference discrete choice experiment. *Health Econ*, 2009; 18:951-976.

16. Petrou S, McIntosh E. (2009) Women's Preferences for Attributes of First-Trimester Miscarriage Management: A Stated Preference Discrete-Choice Experiment. *Value Health*, 12,4:551-559.

17. Hollingworth W, Cohen D, Hawkins J, et al. Reducing smoking in adolescents: cost-effectiveness results from the cluster randomised ASSIST (A Stop Smoking in Schools Trial). *Nicotine & Tob Res*, 2011; 14, 2:161-168.

18.17. Jago, R. Davis, L. McNeil, J. et al. Adolescent girls' and parents' views on recruiting and retaining girls into an after-school dance intervention: implications for extra-curricular physical activity provision. *Int J Behav Nutr Phys Act*, 2011, 8, 91.

19.18. Fordham R, Barton GP. *Promotion of physical activity in children programme guidance: a cost-effectiveness scenario analysis.* London:NICE, 2008.

For peer review only

~~20.~~19. NICE public health guidance 17. *Promoting physical activity, active play and sport for pre-school and school-age children and young people in family, pre-school, school and community settings.* NICE:London, 2009.

~~21.~~20. Hampshire Dance and Laban (2007)[online] Accessed on December 7th 2012. *NRG Youth Dance and Health Report 2005-6.* http://www.hampshiredance.org.uk/documents/NRGEvaluationReport.pdf

~~22.~~21. Bech,M. Gyrd-Hansen,D.. Effects coding in discrete choice experiments. *Health Econ*, 2005; 14,10.

~~23.~~22. STATACORP LP (2009) *STATA user's guide release 11.* Texas, USA: STATACORP LP.

23. Hollingworth W, Cohen D, Hawkins J, et al. Reducing smoking in adolescents: cost-effectiveness results from the cluster randomised ASSIST (A Stop Smoking in Schools Trial). *Nicotine & Tob Res*, 2011; 14, 2:161-168.

24. Safalra [online] 2012. *Historical UK Inflation and price conversion rates.* Accessed on May 3rd 2012 at:- http://safalra.com/other/historical-uk-inflation-price-conversion/.

25. Oanda [online] 2012. *Historical exchange rates.* Accessed on May 3rd 2012 at:- http://www.oanda.com/currency/historical-rates/.

~~26. Fordham R, Barton GP. *Promotion of physical activity in children programme guidance: a cost-effectiveness scenario analysis.* London:NICE, 2008.~~

~~27. NICE public health guidance 17. *Promoting physical activity, active play and sport for pre-school and school-age children and young people in family, pre-school, school and community settings.* NICE:London, 2009.~~

For peer review only

28. Hampshire Dance and Laban (2007)[online] Accessed on December 7th 2012. *NRG Youth Dance and Health Report 2005-6.* http://www.hampshiredance.org.uk/documents/NRGEvaluationReport.pdf

29.26. TES [online] 2011 *Teacher's pay scales from September 2011 unchanged from 2010.* http://www.tes.co.uk/article.aspx?storycode=6000186

30.27. Rush B, Sheill A, Hawe P. A census of economic evaluations in health promotion. *Health Educ Res,* 2004; 19:707-719.

31.28. Kelly MP, McDaid D, Ludbrook A, Powell JE. *Economic appraisal of public health interventions.* London:Health Development Agency, 2005.

32.29. Anderson R. New MRC guidance on evaluating complex interventions. *BMJ,* 2008;337:944-5.

33.30. Buchanan J, Wolstenholme J, Foster C. *A rapid review of economic literature related to the promotion of physical activity, play and sport for pre-school and school-age children in family, pre-school, school and community settings.* London:NICE, 2008.

34.31. Richardson AK. Investing in public health: barriers and possible solutions. *J Public Health* 2012; 34:322-7.

35.32. Ogilvie D, Bull F, Powell J, et al. An applied ecological framework for evaluating infrastructure to promote walking and cycling: the iConnect study. *Am J Public Health*. 2011;101:473-481.

For peer review only

~~36.~~33. Powell J. Compression of morbidity outcomes key to investment in public health. *J Public Health* 2012; 34:329.

~~37.~~34. Alvarez-Farizo B, Hanley N, Barberan R. The value of leisure time: a contingent rating approach, *J Environ Plan Man*, 2001; 44(5):681-699.

~~38.~~35. O'Neill, J. Pate, R. Hooker, SP. The contribution of dance to daily physical activity among adolescent girls. *Int J Behav Nutr Phys Act*, 2011; 8:87.

~~39.~~36. Nader PR, Bradley RH, Houts RM, McRitchie SL, O'Brien M: Moderate-to-vigorous physical activity from ages 9 to 15 years. *JAMA* 2008, 300:295-305.

~~40.~~37. Alvarez-Farizo B, Hanley N, Barberan R. The value of leisure time: a contingent rating approach, *J Environ Plan Man*, 2001; 44,5:681-699.

~~41.~~38. Ridyard C, Hughes D. Methods for the collection of resource use data within clinical trials: a systematic review of the studies funded by the UK Health Technology Assessment Programme. *Value Health*, 2010; 13, 8:867-872.

For peer review only

TABLES

Table I. Attributes and levels of the Discrete Choice Experiment and the four choice sets given to participants

Level of attributes	Attributes		
	Frequency of dance classes per week	Cost per session	Hours left for other leisure activities on that day
**Upper**	2 dance classes per week	£1 per session	Leaving 3 hours for other leisure activities on the evening of the dance session
**Lower**	1 dance class per week	50p per session	Leaving 2 hours for other leisure activities on the evening of the dance session

1A<br>You take one after school dance class each week at a cost of £1 per class leaving you 3 hours on that evening for other leisure activities	1B<br>You take two after school dance classes each week at a cost of 50p per class leaving you 2 hours on those evenings for other leisure activities
2A<br>You take two after school dance classes each week at a cost of £1 per class leaving you 2 hours on those evenings for other leisure activities	2B<br>You take one after school dance class each week at a cost of 50p per class leaving you 3 hours on that evening for other leisure activities
3A<br>You take one after school dance class each week at a cost of 50p per class leaving you 2 hours on that evening for other leisure activities	3B<br>You take two after school dance classes each week at a cost of £1 per class leaving you 3 hours on those evenings for other leisure activities
4A<br>You take two after school dance classes each week at a cost of 50p per class leaving you 3 hours on	4B<br>You take one after school dance class each week at a cost of £1 per class leaving you 2 hours on that

For peer review only

those evenings for other leisure activities	evening for other leisure activities

For peer review only

Table II. **Resources use identification template used to inform BGDP feasibility study**

NRG Youth Dance & Health Project[1]	
	[2]Total Cost £
Project planning work: initial research into existing action research projects	500.00
Lead artist fee -programme design/artist training	800.00
Artists' travel fees – attending training/planning sessions	637.35
Artists' fee	5,806.00
Artists' travel costs	1,515.44
Coach hire – school group for pilot session	562.88
Space hire	254.70
Disclosures/refreshments	77.98
Postage	64.03
Management fee	4,000.00
Staff travel	443.65
Documentation (dissemination advocacy)	269.70
Road-show event – end of project	562.88
Additional schools workshop	151.80
Total 2005-6 prices £	15,203.53
Teacher time for behaviour management (not included in NRG report)[3]	3,300.00
Total 2007-8 prices £ with teacher management	19,427.76
Total 2010-11 prices £	20,600.00

[1]resource use items identified by Hampshire Dance and Laban, NRG Youth Dance and Health Project Evaluation report[20]
[2]assumptions and costing profile produced by Fordham & Barton (2008)[18] for NICE Guidance 17 (NICE,2009)[19]
[3]This item was identified in the NICE report [20] but not ~~included in the NICE~~ cost~~ing~~ed [18 19]

For peer review only

Table III. Description of resources used, unit volumes, ~~and~~ prices and estimated costs* by category by school by pupil in 2010-11 prices

Stage of BGDP	*Description of resources used*	*Number of units*[1]	*Price per unit*[2]	*Cost £ 2010-11 prices*	*Cost stage*
Pre-Programme planning development stage **0**	Lead dance artist consultation and development work	2 days	£500 per day	1000	
	Lead dance artist time, to adapt training programme for hip hop genre	1 day	£500 per day	500	
	Lead dance artist time, to prepare dance teachers for 9 week intervention	1 day	£500 per day	500	
	3 dance teachers preparation/training time	7.5 days	£27 per hour	2400	
	Travel expenses			288[3]	
	Disclosures CRB forms			38[3]	4726
Programme preparation stage **1**	Space hire for dance taster sessions in intervention schools	6 hours	£15 per hour	90	
	Dance teacher delivery of taster sessions in intervention schools	6 hours	£27 per hour	162	
	Control schools (n=4) recruitment presentations	1 day	500 per day	500	
	Postage costs			25[3]	
	Travel expenses			42[3]	819
Programme delivery stage **2**	Delivery 18 dance classes at 90 minutes per class over 9 weeks x 3 schools	81 hours	£27 per hour	2187	
	T-shirts for 3 intervention schools	90 girls	£5 per T-shirt	450	
	Small gift incentives for control schools data collection	3 waves	£731 per wave	2192	
	Refreshments			100[3]	
	Travel expenses			446[3]	5375
Programme reinforcement stage **3**	Half-day dance workshops for two control schools	9 hours	£27 per hour	243	
	6 hour performance events for parents of girls at 3 intervention schools	18 hours	£27 per hour	486	729
				Total	£11,649

BGDP Stages 0-3 costs £s	£11,649 2010-11 prices
BGDP Stages 1-3 costs £s	£6,923 2010-11 prices
BGDP Stages 1-3 minus control costs £s	£3,988 2010-11 prices
BGDP cost per school £s	£1,329 2010-11 prices
BGDP cost per pupil £s	£44.31 2010-11 prices

*Research team administration, travel and other costs are not included. Control costs in this research are included for information

Sources: 1. research team 2. project budget - all prices are actual rates paid. 3. Project budget - all costs are actual expenses incurred.

For peer review only

Resources, estimated total costs and average cost per participant 2010-11 prices

Stage of BGDP	*Description of resources used*	*Total cost $ (£, €) 2010-11 prices*
**Stage 0** Pre-Programme planning development	Nine hours to adapt training programme for hip hop genre, two 3 hour sessions to prepare dance teachers for 9 week intervention delivery by lead dance teacher, 20 hours preparation by 3 dance teachers, disclosures CRB forms	**$8,525** £5,326 €6,238
**Stage 1** Programme preparation	Space hire for two 3 hour dance taster sessions, dance teacher's delivery of two 3 hour taster sessions, 2 control recruitment presentations (90 minutes), postage costs	**$936** £585 €685
**Stage 2** Programme delivery	Delivery of 18 dance classes at 90 minutes per class over 9 weeks in three schools, incentives for intervention schools, incentives for control schools data collection, refreshments	**$9,575** £5,982 €7,007
**Stage 3** Programme reinforcement	Three dance workshops for two control schools at 90 minutes per workshop, three 6 hour end of intervention performance events for parents	**$1,153** £720 €843
BGDP Stages 0-3	**Total cost**	**$20,189** £12,613 €14,773
BGDP Stages 1-3	**Total cost**	**$11,664** £7,287 €8,535
BGDP Stages 1-3	**Total cost minus research costs**	**$7,666** £4,789 €5,609
	**BGDP Cost per participant (N = 90)**	**$85.17** £53.21 €62.32

For peer review only

Table IV. Preference rankings of first choice leisure activities at each time point N (%)

	Time 2		Time 1		Baseline Time 0	
After-school leisure activity	*Ranking*	*N (%)*	*Ranking*	*N (%)*	*Ranking*	*N (%)*
Go around with friends to shopping centres, streets, parks just for fun	1	46 (26)	1	33 (25)	2	12 (18)
Use the internet for fun: chats, YouTube, Facebook, Bebo, Myspace, looking for music (do not include school homework)	2	31 (17)	3	20 (15)	3	8 (12)
Take part in sports, athletics or physical activity	2	31 (17)	2	22 (17)	1	13 (20)
Play with or see friends at your home or their homes	3	21 (12)	4	11 (9)	5	5 (7)
Read books for enjoyment (do not include school books)	4	13 (7)	5	10 (8)	4	6 (9)
Go to discos or dance classes	5	11 (6)	8	5 (4)	5	5 (7)
Play a musical instrument, sing, draw, paint or write	6	9 (5)	4	11 (9)	3	8 (12)
Send text messages or use Twitter on your mobile phone	7	8 (5)	7	6 (5)	5	5 (7)
Play computer games	8	4 (2)	6	8 (6)	6	4 (6)
Watch TV DVDs or playbacks of programmes	8	4 (2)	9	4 (3)	7	2 (3)
Total of valid* responses/total responses		178/210		130/210		68/210

*A valid response = each after-school leisure activity is ranked by a separate number between 1 and 10 by each individual participant using a PDA

For peer review only

Table V. ~~Order of preferences and preference proportions of levels~~Regression coefficients indicating the value of dance classes at t0 (week 0) and t1 (week 9) by group allocation

	Control time 0 (n=104/120)*			*Control time 1 (n=104/120*)*			*Intervention time 0 (n=80/90*)*			*Intervention time 1 (n=80/90*)*		
	Coeff	SE	*P value*	Coeff	SE	*P value*	Coeff	SE	*P value*	Coeff	SE	*P value*
**Frequency of dance class:**			*<0.01*			*<0.01*			*<0.01*			*0.04*
Twice a week	**0.18**	0.07		**0.30**	0.07		**0.25**	0.58		**0.13**	0.06	
Three times a week	-0.18	0.07		-0.30	0.07		-0.25	0.58		-0.13	0.06	
**Cost:**			*<0.01*			*<0.01*			*<0.01*			*<0.01*
£1	**0.22**	0.05		-0.17	0.04		**0.46**	0.07		**0.26**	0.06	
£2	-0.22	0.05		**0.17**	0.04		-0.46	0.07		-0.26	0.06	
**Other hours available for leisure activities on dance class days:**			*<0.01*			*<0.01*			*<0.01*			*<0.01*
2 hours	**0.35**	0.06		-0.31	0.65		**0.76**	0.11		**0.37**	0.08	
3 hours	-0.35	0.06		**0.31**	0.65		-0.76	0.11		-0.37	0.08	

*Preferred level of attribute in bold, attribute most concerned with shaded dark grey, attribute least concerned with shaded light grey *Number of valid responses from total possible responses*

For peer review only

	Level preferences Preference proportions[1]			
Ranking of Attributes	Control time 0 (n=104/120*)	Control time 1 (n=104/120*)	Intervention time 0 (n=80/90*)	Intervention time 1 (n=80/90*)
1. Time for other activities on dance class days<br>2 hrs/day v 3hrs /day	Having 2 hours remaining for other leisure activities on dance class days<br>67% v 33%	Having 3 hours remaining for other leisure activities on dance class days<br>35% v 65%	Having 2 hours remaining for other leisure activities on dance class days<br>82% v 18%	Having 2 hours remaining for other leisure activities on dance class days<br>68% v 32%
2.Cost<br>50p v £1	£1 cost<br>39% v 61%	50p cost<br>58% v 42%	£1 cost<br>28% v 72%	£1 cost<br>37% v 63%
3. Frequency of dance classes per week<br>1 p/wk v 2 p/wk	2 classes per week<br>41% v 59%	2 classes per week<br>36% v 64%	2 classes per week<br>38% v 62%	2 classes per week<br>44% v 56%

[1] Preference proportions holding all else equal, the proportion of the sample that would choose particular level over the other within that attribute
*Number of valid responses from total possible responses

For peer review only
